# Reconstruction of a 10-mm-long median nerve gap in an ischemic environment using autologous conduits with different patterns of blood supply: A comparative study in the rat

**DOI:** 10.1371/journal.pone.0195692

**Published:** 2018-04-16

**Authors:** Diogo Casal, Eduarda Mota-Silva, Inês Iria, Sara Alves, Ana Farinho, Cláudia Pen, Nuno Lourenço-Silva, Luís Mascarenhas-Lemos, José Silva-Ferreira, Mário Ferraz-Oliveira, Valentina Vassilenko, Paula Alexandra Videira, João Goyri-O’Neill, Diogo Pais

**Affiliations:** 1 Anatomy Department, NOVA Medical School, Universidade NOVA de Lisboa, Lisbon, Portugal; 2 Plastic and Reconstructive Surgery Department and Burn Unit, Centro Hospitalar de Lisboa Central–Hospital de São José, Lisbon, Portugal; 3 UCIBIO, Life Sciences Department, Faculty of Sciences and Technology, Universidade NOVA de Lisboa, Caparica, Portugal; 4 CEDOC, NOVA Medical School, Universidade NOVA de Lisboa, Lisbon, Portugal; 5 LIBPhys, Physics Department, Faculdade de Ciências e Tecnologias, Universidade NOVA de Lisboa, Lisbon, Portugal; 6 Pathology Department, Centro Hospitalar de Lisboa Central–Hospital de São José, Lisbon, Portugal; University of Sydney, AUSTRALIA

## Abstract

The aim of this study was to evaluate in the Wistar rat the efficacy of various autologous nerve conduits with various forms of blood supply in reconstructing a 10-mm-long gap in the median nerve (**MN**) under conditions of local ischemia. A 10-mm-long median nerve defect was created in the right arm. A loose silicone tube was placed around the nerve gap zone, in order to simulate a local ischemic environment. Rats were divided in the following experimental groups (each with 20 rats): the nerve Graft (**NG**) group, in which the excised MN segment was reattached; the conventional nerve flap (**CNF**) and the arterialized neurovenous flap (**ANVF**) groups in which the gap was bridged with homonymous median nerve flaps; the prefabricated nerve flap (**PNF**) group in which the gap was reconstructed with a fabricated flap created by leaving an arteriovenous fistula in contact with the sciatic nerve for 5 weeks; and the two control groups, Sham and Excision groups. In the latter group, the proximal stump of the MN nerve was ligated and no repair was performed. The rats were followed for 100 days. During this time, they did physiotherapy. Functional, electroneuromyographic and histological studies were performed. The CNF and ANVF groups presented better results than the NG group in the following assessments: grasping test, nociception, motor stimulation threshold, muscle weight, and histomorphometric evaluation. Radial deviation of the operated forepaw was more common in rats that presented worse results in the other outcome variables. Overall, CNFs and ANVFs produced a faster and more complete recovery than NGs in the reconstruction of a 10-mm-long median nerve gap in an ischemic environment in the Wistar rat. Although, results obtained with CNFs were in most cases were better than ANVFs, these differences were not statistically significant for most of the outcome variables.

## Introduction

Although bold surgeons such as Paul of Aegina were reportedly performing nervous sutures 600 AD, even today, despite numerous surgical and technical developments, the results with peripheral nerve repair are still disappointing.[1–4] Results are particularly unsatisfactory in cases of long nerve defects, being frequent not to obtain useful recovery in the involved nerve territory.[1–3] In 1870, Philipeaux and Vulpian proposed the use of nerve grafts (**NGs**) (devoid of intrinsic blood flow until neoangiogenesis from neighboring tissues occurs) to promote axonal regeneration through nerve defects.[5] Since then, NGs have become the gold standard for the reconstruction of peripheral nerve defects.[6] Even today, the multiple modern techniques of tubulization, using artificial nerve conduits, are generally discouraged for reconstructing defects over 5 to 6 cm in length.[1–3, 7–10] Additionally, experimental data suggests that autologous NG may yield superior motor recovery compared to nerve conduits, and to processed nerve allografts.[11]

From their inception, it was realized that the results obtained with NGs were far from perfect. Consequently, in 1921, Ney proposed the use of vascularized nerve segments, based on a conventional blood supply with an arterial and venous blood supply.[12] These conventional nerve flaps (**CNF**s) were further developed by Strange and Seddon in 1947.[13, 14] However, all these authors described pedicled CNFs, whose usefulness was limited because they could only be mobilized locally. Only in 1976 was the concept of free CNF introduced by Taylor.[15] Still, these free CNFs are based on very small-sized nourishing vessels, making their dissection laborious and the vascular anastomoses at the recipient site difficult. Additionally, due to anatomical variations, they cannot always be raised.[16] Finally, there is a limited number of available CNFs.[17–19]

In order to circumvent these problems, in 1984, Taylor and Townsend proposed the use of arterialized neurovenous flaps (**ANVFs**).[20] These nerve flaps are based on the anatomical proximity of the venous system to multiple nerves, particularly in the subcutaneous tissue.[21, 22] ANVFs are thus composed of expendable nerve segments and adjoining veins.[21] Usually, one end of the vein is anastomosed to a recipient site artery and the other end is connected to a recipient site vein.[22] ANVFs can be harvested easily from multiple places of the body, particularly from the limbs, having a relatively expedite dissection. Furthermore, superficial veins have a large enough caliber to allow relatively easy vascular anastomoses.[20] Occasionally, the vascular architecture of ANVFs can be used to simultaneously reconstruct adjacent vascular and nerve defects.[22]

CNFs seem to guarantee better functional results than NGs for bridging nerve defects, particularly in conditions of local ischemia or fibrosis, as they are less likely to undergo central necrosis and histological disorganization.[23–25] In fact, contrarily to NGs, CNFs do not depend on plasmatic imbibition during the first 3 to 4 days after transfer for survival.[25–30]. Other authors argue that nerve flaps are not necessarily advantageous, as nerve grafts rapidly regain a new blood supply in several experimental models.[31] Some authors add that, although recovery tends to be faster with CNFs, the end functional results are similar with CNFs and NGs.[25, 31]

Currently, as far as the authors could determine, the evidence of the efficacy of ANVFs for bridging nerve defects is limited to two articles on the reconstruction of femoral nerve defects in the rat.[29, 30]

Cavadas *et al*. suggested prefabrication of nerve flaps by placing an arteriovenous fistula in contact with nerve segments.[32] Theoretically, this could allow the creation of nerve flaps in virtually any place of the body, solving many of the problems with CNFs. Additionally, there is one study reporting that these prefabricated nerve flaps (**PNFs**) present superior results than NGs in the reconstruction of nerve defects in conditions of local compromise of circulation.[33] Yet, PNFs are not routinely used in clinical practice, in great part due to lack of supporting evidence of their usefulness.[34]

One of the reasons why conclusive evidence is difficult to obtain in the realm of peripheral nerve gap reconstruction is that different researchers have used different animal species, anatomical regions, reconstructive strategies and follow-up times. Furthermore, authors have also used variable outcome variables to assess nerve regeneration. These methodological differences make information synthesis challenging.[31] Finally, even though there are a few side-to-side comparisons of different gap reconstruction methods in the rat hindlimb, using the sciatic nerve, as far as the authors could determine, there is no similar study using the rat forelimb.[11, 23, 35] This is unfortunate, since clinically most peripheral nerve lesions occur in the upper extremity.[3, 36]

Therefore, the aim of this study was to evaluate in the Wistar rat the efficacy of various autologous nerve conduits with various forms of blood supply in reconstructing a 10-mm-long gap in the median nerve (**MN**) under conditions of local ischemia.

## Methods

### Animal well-being and ethical committee’s approval

All *in vivo* studies involving rats were carried out in strict accordance with or exceeding the recommendations in the Guide for Proper Conduct of Animal Experiments and Related Activities in Academic Research and Technology.[37, 38]

The experimental protocol was approved by the Institutional Animal Care and Use Committee and Ethical Committee at the authors’ institution (CEFCM/08/2012).

### Pre-operative training and accommodation

Three weeks before the surgery, rats were accustomed to being handled by the researchers. In addition, they were familiarized with the different functional tests used in the postoperative assessment that are described below. During this period the rats were manipulated daily.[39, 40] Pre and post-operatively the rats were maintained in an enriched environment. They were kept in customized cages of 60 X 30 X 90 cm, each with four stages, three ladders, a suspended rope and a training wheel. Each cage contained 5 to 6 rats. This environment intended to mimic the usual physiotherapy that peripheral nerve patients are typically offered postoperatively.[41]

Rats were individually identified by an ear notching and marking system, under general anesthesia.[42]

### Perioperative care of experimental animals

All the animals were housed under standard environmental conditions and fasted six hours before surgical procedures. No antibiotic prophylaxis was given.

Rats were anesthetized with a mixture of ketamine (5 mg/kg) and diazepam (0.25 mg/kg) given intraperitoneally. The depth of anesthesia was evaluated by toe pinch and by observance of respiration rate throughout the entire procedure. Supplementary doses of the anesthetic solution were provided as needed.[43]

After shaving the surgical sites and placing the animals on the operation table, the surgical field was disinfected with an antiseptic solution (Cutasept®) and draped. All surgical procedures were performed under strict antiseptic procedures. Surgeries were performed by the same author (D.C.), in order to avoid inter-surgeon variability. Surgical procedures were performed under a stereotaxic operating microscope (Leica® M651) and using microsurgical instruments. Hypothermia was avoided by placing the rat over a heating pad during surgical procedures and in the postoperative period.

### Surgical model of nerve gap and ischemia in the rat’s forelimb

**Fig 1:**
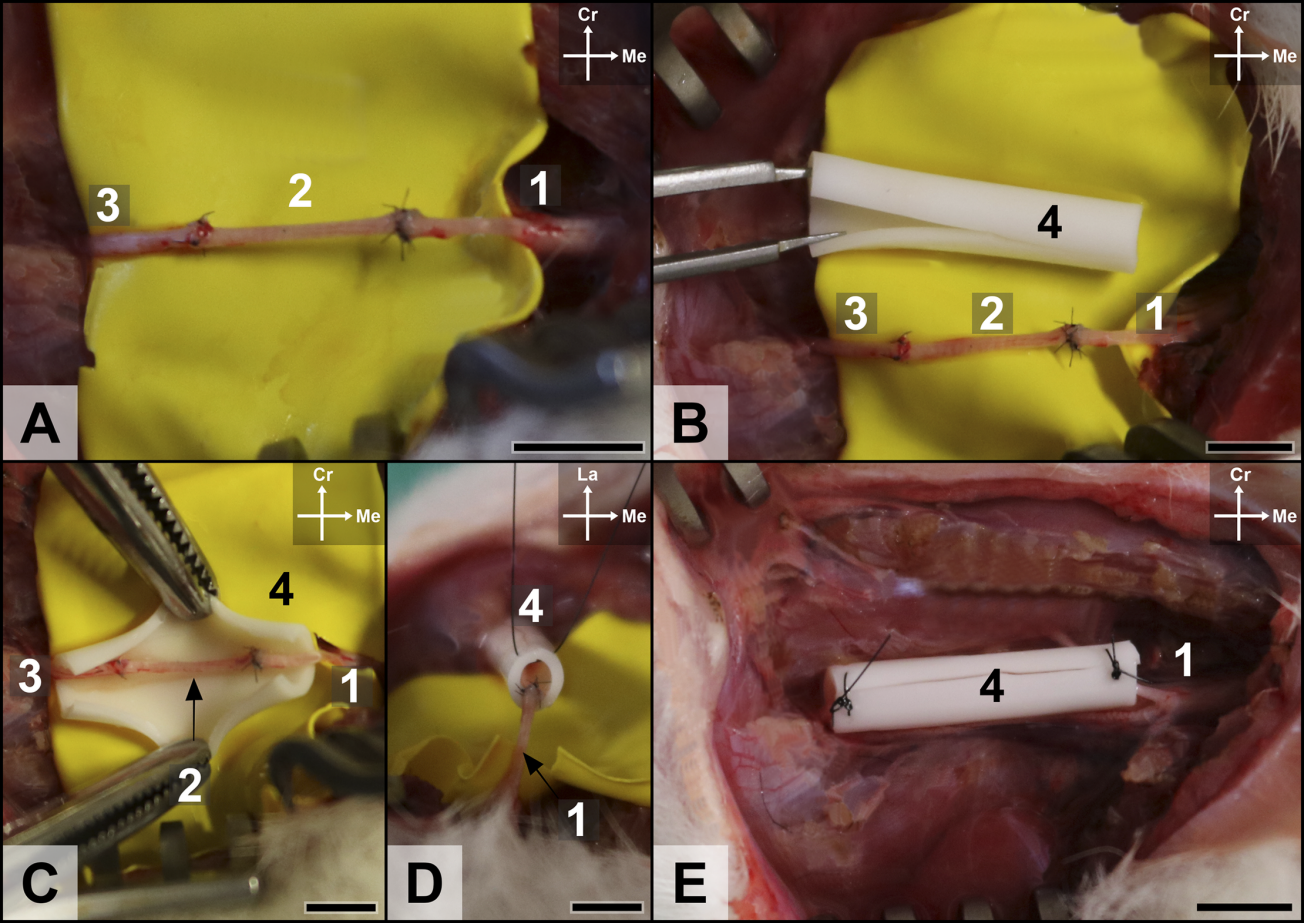
Representative intraoperative photographs of the model used to simulate ischemia surrounding the median nerve gap in the right forepaw of a rat in the nerve graft experimental group. (A) The nerve defect bridged with the autologous inverted median nerve graft.(B and C) A silicone rod is longitudinally opened and placed around the median nerve reconstruction. (D) The silicone sheath is secured with interrupted 6–0 Nylon stitches places at both ends. (E) Final appearance of the nerve reconstruction site before wound closure.1, proximal stump of the median nerve; 2, autologous median nerve graft; 3, distal stump of the median nerve; 4, silicone rod place around the nerve gap to simulate an ischemic environment.Cr, Cranial; Me, Medial. Calibration bar = 1 cm.

**Fig 2:**
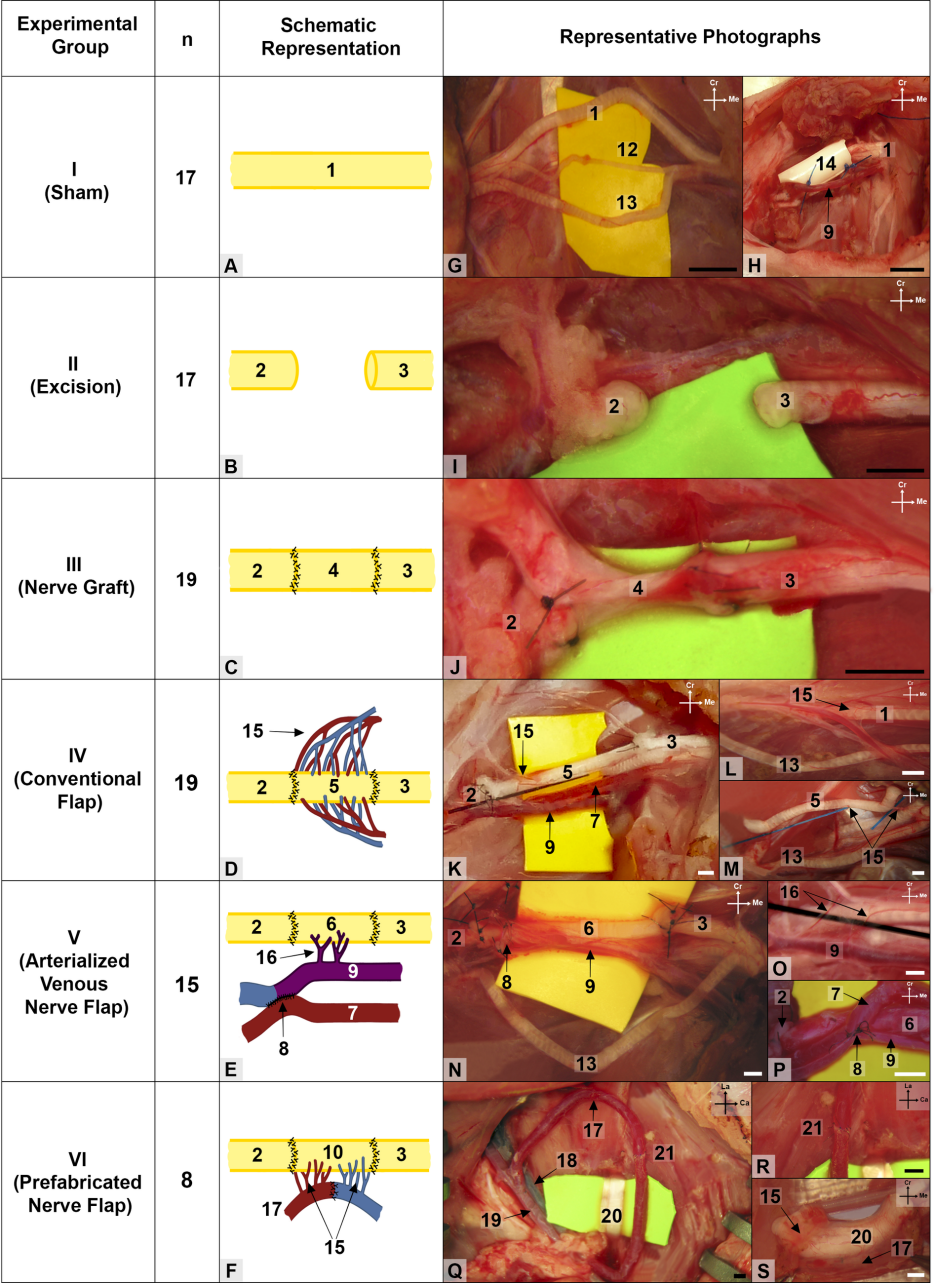
Experimental groups’ schematic representation and representative photographs. (A to F) Schematic drawings of the different methods of bridging the median nerve gap in the various experimental groups. (G to S) photographs of representative intra-operative images. All images represent the right forelimb with the exception of (Q) which represents the left groin region. 1, median nerve; 2, distal stump of the median nerve; 3, proximal stump of the median nerve; 4, autologous median nerve graft; 5, median nerve conventional flap; 6, arterialized neurovenous flap; 7, brachial artery; 8, arterio-venous anastomosis; 9, brachial vein; 10, prefabricated nerve flap; 11, arterio-venous fistula used to produce the prefabricated nerve flap; 12, medial antebrachial nerve; 13, ulnar nerve; 14, silicone rod place around the nerve gap to simulate an ischemic environment; 15, *vasa nervorum* to median nerve flap; 16, *vena nervorum*; 17, arteriovenous loop; 18, femoral vein; 19, femoral artery Ca, Caudal; Cr, Cranial; La, Lateral; Me, Medial. Calibration bar = 1 mm.

A surgical model of ischemia surrounding a 10-mm-long median nerve defect was used (**Fig 1**).[30, 40, 44–46] As other authors, we have used a loose silicone tube with a 5-mm-wide inner diameter, and of 15-mm-length (Fortune Medical Inst. Corp.®; Reference 2011–0035) around the nerve repair zone, in order to simulate a local ischemic environment.[30, 44] This tube was sectioned longitudinally, so that it could be opened and subsequently be used to isolate the region of the reconstructed nerve segment (**Fig 2H**). When vascularized nerve segments were used, the longitudinal opening in the silicone tube was left wide enough to accommodate the *vasa nervorum* supplying the nerve conduit, while isolating the reconstructed nerve segment from the surrounding vascularized tissues. At the end of the procedure a simple 6–0 Nylon stitch (Dermalon® Suture 6–0 Nylon C-1 Blue 18" Monofilament 36 Cutting) was placed at each end of the tube, passing both sides of the slit, to prevent migration of the silicone tube.

One hundred and twenty female Wistar rats, aged 4 to 6 months, and weighing between 200 and 250 grams, were randomly allocated in one of the following experimental groups in equal numbers (n = 20):

#### Sham group

a longitudinal incision was made until the subfascial plane throughout the entire medial aspect of the right arm. A myotomy of the lateral portion of the sternal head of the pectoralis major muscle was then performed, in order to expose the MN. This nerve was gently teased away from the surrounding structures in the arm region, becoming pedicled in its segmental blood supply from the brachial vessels.[47] The mentioned 15-mm-long silicone tube was placed around the nerve preserving its local feeding branches. The skin wound was sutured with interrupted 5–0 Nylon (5–0 Ethilon® Black 18" Fs-2 Cutting) stitches in all the experimental groups (**Fig 2A, 2G** and **2H**).

#### Excision group

After exposing the right median nerve as detailed above, a 10-mm-long segment in the central portion of the MN in the arm was marked with a surgical ruler and a surgical marker. The region of the median nerve proximal and distal to this marked region was tagged with 10–0 Nylon (10–0 Ethilon® Black Monofilament 2870G Round Body Taper Point) stitches. The marked region was cut sharply with a pair of straight microsurgery scissors. The excised segment was discarded. The proximal stump of the median nerve was ligated with an 8/0 Nylon stitch (8–0 Ethilon® Black 5" Bv130-5 Taper). The stumps of the median nerve were placed inside both endings of the silicone tube (**Fig 2B** and **2I**).[48]

#### Nerve graft (NG) group

Following the excision of the 10-mm-long segment of the right MN as described above, the nerve segment was placed in its original. The nerve segment was not placed in an inverted position, as it is normally recommended, to facilitate comparison with the remaining experimental groups (**Fig 2C** and **2J**). Nerve repair was performed using four to six interrupted 10–0 epineural Nylon stitches (10–0 Ethilon® Bv75-3 Taper) in all the experimental groups using conduits.[49]

#### Conventional nerve flap (CNF) group

The MN of the rat at the arm level possesses a type A blood supply, that is to say it is an unbranched nerve supplied segmentally by the median artery and vein which are in parallel.[16, 47] To produce a CNF of the MN, the 10-mm-long nerve segment was carefully dissected and excised as detailed above, leaving it pedicled solely on its epineural arteries and veins in the brachial region (**Fig 2D, 2K, 2L** and **2M**).[44]

#### Arterialized neurovenous flap (ANVF) group

After isolating and cutting the 10-mm-long nerve segment as described for the CNF group, all epineural arteries were carefully cauterized, leaving the nerve conduit pedicled solely on the epineural veins in the brachial region. Immediately proximally to the terminal division of the brachial artery, the brachial artery and accompanying vein were anastomosed lateral-laterally after performing a 1.5-mm-long incision the adjoining flanks of these two vessels. The anastomosis was performed with six to eight interrupted 12–0 Nylon stitches (S&T®; 50 μm needle; Ref. 03194). Consequently, an arterial venous anastomosis was created in the distal aspect of the arm, leading to the creation of an arterialized neurovenous conduit, which was used to bridge the MN gap (**Fig 2E, 2N, 2O** and **2P**).

#### Pre-fabricated nerve flap (PNF) group

**Fig 3:**
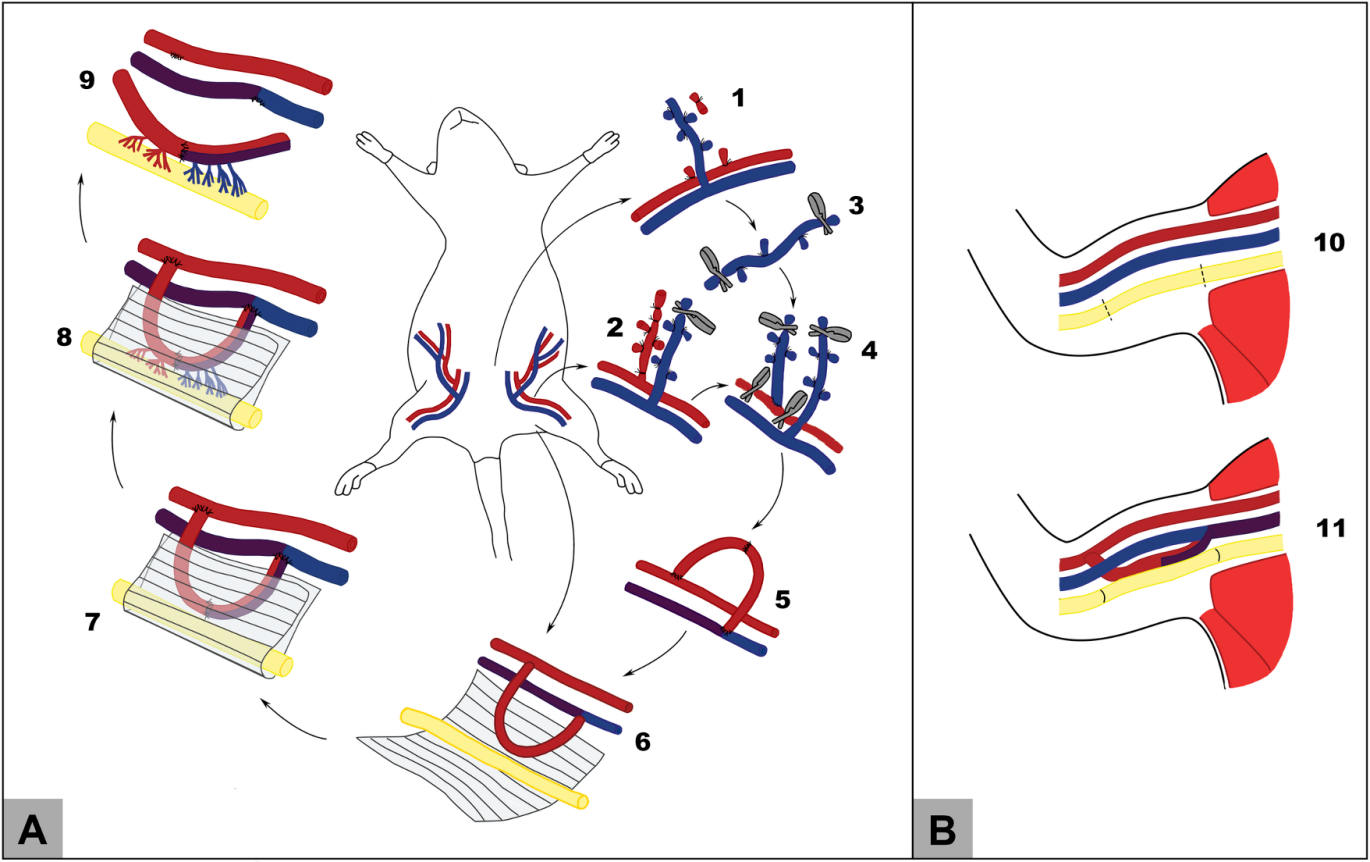
Conventional flap pre-fabrication and transfer. (A) Prefabrication of the flap in the left thigh. (B) Insetting of the flap in the recipient area in the right arm. 1. An inverted “T” incision is performed in the most caudal aspect of the ventral region of the abdomen of the rat, with the axial portion crossing immediately cranial to the pubic symphysis and with the longitudinal component extending from this point cranially for 3 cm. 2. The right superficial caudal epigastric vein is dissected from the homonymous artery and the caudal epigastric nerve during its entire length, including also part of the its lateral afferent vein. 3. The venous segment is harvested and its origin and termination sites are ligated with interrupted 9/0 Nylon stitches. 4. The venous conduit is inverted and its terminal-laterally anastomosed to the left femoral artery using an interrupted 11/0 Nylon suture. 5. The two epigastric veins are terminal-terminally anastomosed with interrupted 11/0 Nylon stitches, producing and arterial-venous fistula; 6. The left sciatic nerve is exposed through a ventral approach in the medial aspect of the thigh, in the space between the gracilis muscle, placed laterally, and the semimembranosus muscle, located medially. The medial femoral circumflex vessels are ligated and divided. The arterial-venous fistula is placed over the ventrally exposed left sciatic nerve. A silicon sheath is placed around the nerve and the arterial-venous fistula. 7. The silicone sheath is folded on itself and maintained in place with interrupted 5/0 Nylon stitches. The surgical wounds are closed with interrupted 5/0 Nylon stitches. 8. The sciatic nerve and fistula are maintained in contact for 5 weeks, allowing the development of vascular connections between the fistula and the sciatic nerve. 9. After five weeks, a conventional flap including a segment of the sciatic nerve measuring approximately 15 mm has been fabricated. 10. A 10-mm-long segment of the right median nerve is excised. 11. The prefabricated nerve flap is inset in the region of the median nerve defect. Excessive neural tissue is trimmed at both ends. The arterial end of the arterial-venous fistula was terminal-laterally anastomosed to the distal portion of the brachial artery and the venous end of the fistula was terminal-laterally anastomosed to the proximal aspect of the brachial vein using interrupted 12/0 Nylon stitches. Neural anastomoses were performed using interrupted epineural 11/0 Nylon sutures.

In this group, a conventional nerve flap was fabricated around the left sciatic nerve of the rat using the technique described by Cavadas and Vera-Sempere (**Fig 2F, 2Q** and **2R**).[32] Succinctly, an arterial-venous fistula was created in the ventral aspect of the left thigh using the superficial caudal epigastric veins, which were connected to the femoral artery (**Fig 3**). The anastomosis between the superficial caudal epigastric veins was performed with four to six interrupted 12–0 Nylon stitches (S&T®; 50 μm needle; Ref. 03194). The anastomosis between the proximal superficial caudal epigastric vein and the femoral artery was achieved with resort to eight to ten 11–0 simple Nylon stitches (Ethilon® Black 5" Bv50-3). The arterial-venous fistula was maintained in contact with the left sciatic nerve for 5 weeks. This lead to the fabrication of a conventional perfusion flap including a segment of the left sciatic nerve. Subsequently, this PNF was transferred to the right arm to reconstruct the median nerve defect (**Fig 2F, 2Q** and **2R**; and **Fig 3**). The arterial end of the arterial-venous fistula was terminal-laterally anastomosed to the distal portion of the brachial artery and the venous end of the fistula was terminal-laterally anastomosed to the proximal aspect of the brachial vein using interrupted 12–0 Nylon sutures (S&T®; 50 μm needle; Ref. 03194) (**Fig 3B**).

### Postoperative evaluation

Rats were assessed daily regarding general activity, grooming, signs of wound infections or dehiscence, as well as for evidence of autotomy.[50–52] In case of significant wound complications, signs of pain and/or distress, autophagy and/or self-mutilation of the operated limbs, rats were euthanized under general anesthesia by exsanguination.[53, 54] Experimental animals were maintained in the recommended cycles of light and darkness. Animals were provided food and water *ad libitum*.[41]

Rats were followed for 100 days (**D**) after MN reconstruction.

Every 15 days (D15, D30, D45, D60, D75, D90), they were submitted to the following evaluations: grasping test; nociception evaluation; running velocity; walking track analysis. On D90, after being subjected to these evaluations, they were anesthetized as described above, and submitted to injection of retrogradely labelling neuronal markers. On D100, rats were submitted to infra-red thermography (**IRT**) of the palmar aspect of the forepaws, electroneuromyography (**ENMG**), and strength evaluation after direct MN stimulation. Subsequently, a median ventral thoracotomy was performed, and the left and right ventricles were catheterized with 20G silicone catheters. The rats were submitted to exsanguination and replacement of blood volume by heparinized saline injected through the left ventricle, followed by 300 ml of 4% paraformaldehyde in 0.1M PBS (pH 7.40).[55] Finally, after a 24h incubation period, both flexor carpi radialis muscles were harvested from their origin in the medial epicondyle until their insertion on the palmar aspect of the carpal and metacarpal regions and immediately weighted using a precision scale (Kern® 770).[46] Nerve tissue was also collected for histomorphometrical analysis using conventional stains, immunohistochemistry and fluorescence microscopy.[46]

#### Grasping test

This test was used to assess motor recovery of the muscles controlled by the MN.[39] To ensure consistency and reproducibility of the grasping test, the authors performed this test using the widely-accepted methodology suggested in Bertelli’s original description.[39] However, contrarily to this description, the authors did not immobilize the contralateral paw, to prevent undue stress.[56, 57] All the evaluations were performed by a trained observer, blinded to the experimental group, starting one month prior to the beginning of the experiment, in order to facilitate the rats’ adaptation. The rat was suspended by its tail over a grid, which it reflexively tried to grab. The animal was gently pulled by its tail with increasing strength until it loosened its grip. The rat was able to grab the grid only if the MN was functioning. Grasping strength was graded, in a similar fashion to that recently described by Stöβel *et al*.[57], as follows: 0 –no grasping movement; 1- slight flexion of fingers, but with no significant grasping strength; 2- minimal grasping strength; 3- significant grasping strength but still inferior to the unaffected contralateral side; 4- normal grasping strength (equal to the contralateral, non-affected, limb).[39, 56]

#### Pin prick test

This test was used to evaluate nociception.[50, 58] In this test, the rats were placed on an elevated plastic platform with a 4x4 mm square grid pattern with 1.9 mm of length each. This grid was supported by a metallic frame that was 21-cm-tall. The grid was covered with a transparent plastic box with the following dimensions of 15.5x15.5x11cm.[50, 58] For each rat and evaluation point, rats were left on the platform covered by the plastic box for a few minutes until the exploratory and major grooming activities subsided. Subsequently, a number 4 Von Frey hair (bending force of 25g) was inserted through the mesh to poke the palmar aspect of the forepaw in its radial aspect, corresponding to the skin territory of the MN. The evaluation was considered correct only if the Von Frey filament bended.[59] Forepaws were evaluated in turn, when the rat was stationary and standing on the four paws. A few seconds mediated each evaluation to minimize apparent behavioral responses to the previous stimulus. Ambulation and biting the filament were considered ambiguous responses, and in such cases the stimuli were repeated. Five measurements were made for each paw. Each time the following score was used: 0 –no response; 1 –the rat slowly takes the paw away from the Von Frey hair; 2 –the rat vigorously removes the forepaw from the Von Frey hair and/or licks the paw. Consequently, for each rat and time point, a nociceptive score was calculated as the sum of the responses to the five stimuli. This originated a value that ranged from 0 (no response for all noxious stimuli) to 10 (high response for all noxious stimuli).[40, 48, 50, 60–63]

#### Ladder rung walking test

This test was used to assess forelimb strength, stepping, placing, and co-ordination.[64] Rats were trained to run an inclined ladder of 120x9x2cm dimensions with 18 steps, of 1.5-cm-thickness and spaced 4 cm. The ladder was positioned with and inclination of approximately 10 degrees and led to a 13.20x11cm opening on a dark wooden box with 31.5x35x35cm of internal dimensions.

The rats were conditioned to run the ladder and enter the dark box on several training sessions that consisted of 5 trials each. In the first trials, the rats were positioned close to the box’s door and guided in. For the subsequent 3 sessions, rats were progressively positioned further away from the box opening in each trial and persuaded to get in the box by gentle touching and/or pulling of tail’s tip. Once inside the box, the sliding door closed the entrance and the rat was given a food treat. For the last trials, rats would only receive a snack, if they walked through the ladder without stopping or hesitating. Finally, for the last 5 sessions performed before surgery, the time to complete the task was recorded. The examiner started the timer (precision of 1/100sec JUNSD®) once the animal started walking at the beginning of the ladder and stopped the timer when the rat’s snout crossed the box’s entrance. The test was considered valid, if the animal did not stop and did not hesitate during the task. After surgery, each evaluation session consisted of five trials, each separated by at least a one-minute interval. The time taken to complete each run was recorded.[46, 50, 58, 65]

#### Walking track analysis

This test was used to evaluate forelimb motor recovery.[66, 67] The experimental apparatus consisted of a confined walkway with 16.5 cm in height, 8.7 cm in width and 43 cm in length. This walkway lead to a rectangular opening with 8.8x8.2cm in one of the walls of a black wooden box with the dimensions of 23x36x28 cm. The box’s entrance could be closed rapidly by a vertical sliding door. The box had a removable top that could be used to retrieve the rat. [66, 67]

Before the surgery, rats were trained to walk through the walkway until reaching the inside of the box. Particular attention was given to familiarize the rats with the noise of closing the sliding door. To positively condition rats, a food treat was given once the task was completed successfully. For the evaluations, the floor of the walkway was paved with graph paper (Ambar®). Rats forepaws were stained with methylene blue 1% W/V (Merck ®) with a painting brush. The rats were then led into the corridor. This test was done on every evaluation day and repeated as many times as needed until a representative print of both forepaws was obtained. [66, 67]

**Fig 4:**
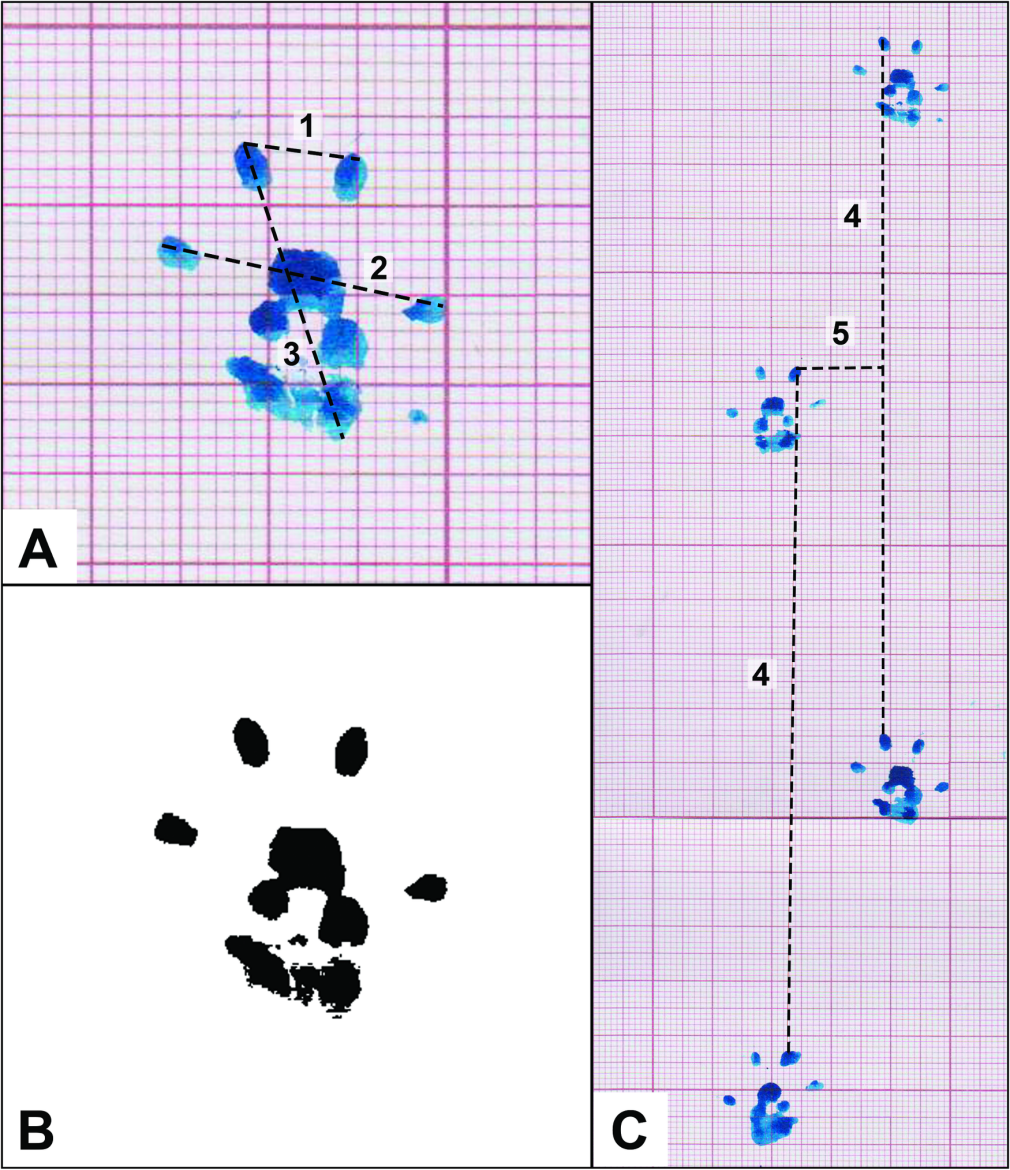
Walking tracks measurements using forepaw impressions. **(A)** Photograph of a typical print of the left forepaw (uninjured). **(B)** Contrast-enhanced image of the photograph in Fig 3A, using the software Fiji®. Similar images were used for measurement purposes, namely of determination of the stance factor (paw impression area on the paper sheet). **(C)** Typical forepaw prints of a rat in the nerve graft group. 1, Intermediate finger spread factor: widest width between the second and third fingers; 2, Finger spread factor: widest width of the paw impression; 3, Print length factor: longest length of the paw impression; 4, Stride length: distance between homologous points of successive paw impressions on a given side; 5, Base of support: perpendicular distance between the central portion of the paw impression and the direction of movement.

The following parameters were assessed for typical consecutive imprints of both forepaws (**Fig 4**) [66]:

**Stance factor:** paw impression area on the paper sheet.

**Print length factor:** longest length of the paw impression.

**Finger spread factor:** widest width of the paw impression.

**Intermediate finger spread factor:** widest width between the second and third fingers.

Additionally, the following parameters were assessed in the two pairs of representative sequential bilateral paw impressions (**Fig 4**) [66]:

**Stride length:** distance between homologous points of sequential paw impressions on a given side.

**Base of support:** perpendicular distance between the central portion of the paw impression and the direction of movement.[40, 50, 66]

Typical morphological patterns of the forepaw impressions were also searched.

Walking track analysis parameters were measured using the free software FIJI®.

#### Infra-red thermography (IRT) of the cutaneous territory of the median nerve

Thermography was used as a non-invasive surrogate marker of cutaneous denervation in the territory of the MN.[68–70] This assessment method was performed in the plantar territory of the MN on D100 after anesthetizing the rat.

The following aspects were taken into consideration before performing IRT:

- A room with a constant temperature, between 18°C to 25°C and without significant heat sources (such as computers or refrigerators). The room temperature and humidity were registered using a normal digital hydro-thermometer (TFA®) with a thermal resolution of 0.1°C.
- The animals were brought to the room where the acquisitions were going to be performed 2 hours prior to the evaluation to allow acclimatization to occur. After being anesthetized, rats were placed on a clean and stable surface away from reflective materials and other possible sources of artefacts. The rat’s central temperature was also monitored during all evaluation using a digital thermometer (Electro® DH SA) with a thermal resolution of 0.1°C inserted 2 cm inside the rectum.

The temperature was assessed using a FLIR® E6 camera, which has an accuracy of ± 2°C within the ambient temperature range and a thermal sensitivity of <0.06°C. An IR resolution of 160 x 120 pixels interpolated on 320 x 240 resolution within the camera electronics. The camera was switched on 15 minutes before acquisition and was not switched off during the experiment. The emissivity parameter was set on the camera for that of the skin, ε = 0.98.

Each rat was gently laid on its dorsum on a polyethylene sponge, and its forepaws carefully fixed in supination with double face glue tape. After 3 minutes, the camera was held at 90° angle and 30 cm distance from the rat, focusing the animal’s body on the camera. Then, three acquisitions were made, spaced 30 seconds apart. In the end, the rectal thermometer was removed.

The acquired thermograms were transferred to a computer and analyzed using the free software FLIR Tools+ ®. The temperature of the plantar surface of both forepaws was measured defining a rectangular region of interest of 9 X 11 pixels in the plantar territory of the MN. The mean, maximum and minimal temperature values were exported to a “.CSV” document and later added to an Excel (Microsoft Office®) and SPSS 21.0 (IBM Statistics®) databases.

#### ENMG

This assessment was performed on D100 on both forelimbs. The authors ensured that rats were deeply anesthetized before starting the acquisition, to minimize variability associated with voluntary and/or involuntary movements autonomously produced by the experimental animal.[71] The evaluations were always performed by the two senior authors (D.C. and E.M.S), in the same room, and under the same controlled environmental conditions.[8, 71, 72]

The experimental setting was composed of BIOPAC MP35® hardware and a BSLM Stimulator®. The electrodes used for stimulation and recording were made by taping a pair of disposable acupuncture needles (0.25x25 mm Shenzhou® acupuncture needles; which had a negligible impedance [<1 ohm]) with a distance of 25 mm between them. The compound muscle action potentials (**CMAPs**) were recorded using the BSL PRO 3.7® software, adjusting settings to create an optimized template. The stimulator and electrodes were connected to the BIOPAC MP35®. The channels were set up as follows: CH1—Stimulator BSLSM to 0-10Volts); and CH2—EMG to 30–1000 Hz. The signal was acquired at a sample rate of 50 kHz, at a duration of 40.000 ms, amplified 1000X and filtered using a 30–1000 Hz band. The stimulation output was set for a single pulse with a duration of 1 ms.[8, 71, 72]

Under the surgical microscope and with the rat in dorsal decubitus, the MN was dissected free from the silicone rod and from the surrounding tissues. The flexor digitorum sublimis muscle was exposed on both forearms with a ventral longitudinal incision. The signal ground plug was connected by inserting the ground needle in the quadriceps femoris muscle of the left hindlimb. Starting with the right forepaw, the recording electrodes were put inside the flexor digitorum sublimis muscle belly and the stimulation electrode held in contact with the MN proximally to the lesion site. Both electrodes were moistened with saline (Basi®). Initially, a stimulation amplitude of 10 mV was chosen by adjusting the stimulator knot and the CMPA was recorded. During the evaluation, the amplitude of stimulation was increased gradually in 10 mV steps until reaching 2 V. The same procedure was then repeated on the left forepaw.[8, 71, 72]

The Biopac analysis software BSL Pro 3.7® was used to quantify the following parameters: neurological stimulation threshold; motor stimulation threshold; latency; neuromuscular transduction velocity; CMAP amplitude; and CMAP duration.[72–74]

#### Wrist flexion strength assessment

As wrist flexion is predominantly dependent on the MN, this evaluation was used on D100 to assess strength in this nerve’s territory. To assess wrist flexion strength the mentioned BIOPAC MP35®, BSLMA stimulator software® and stimulation electrodes were used to stimulate the MN. Using BSL PRO 3.7® software, the following parameters were adjusted to create a template for stimulation: the input channel CH1 was set as Stimulator-BSLSTM (0–10 Volts) and the output settings were selected for stimuli duration of 30 seconds with pulses of 1 ms duration and 1 Hz frequency. The amplitude of the pulses was adjusted on the stimulator knot for 1.5 V or 3 V according to the evaluation moment. A dynamometer, Sauter® FH 5, with a resolution of d = 0.001 N was linked to a computer. The AFH-01® software was installed on the computer and linked to FH 5 dynamometer allowing real time visualization of data by building a plot of force per time (N/sec). This data was later imported to an excel sheet (Microsof Office®) for analysis.[65]

With the rat anesthetized, a silk 5/0 stitch (5–0 Silk Black 18" P-3 Cutting; Ethicon®) was passed through the second interosseous space. This stitch was associated with a 5-cm-long loop. The rat was put on its back and a self-retaining retractor used to expose the nerve. Starting with the right forepaw, the suture loop was placed in the dynamometer’s hook and the forepaw aligned with the dynamometer, without putting too much strain on the suture line. The contralateral paw was fixed with tape to avoid extra movement interferences in the dynamometer readings. The stimulating electrodes were put proximally in the MN and wetted with saline (Basi®). The dynamometer was set to zero and the stimulator adjusted to a supramaximal amplitude stimulation of 1.5 V for 30 seconds. The same steps were done on the left forepaw. The data thus recorded with the AHF1 software® were imported into an Excel® (Microsoft Office™) datasheet. Maximum and average force values and the area under the curve (AUC) for the strength X time graph were calculated for each evaluation.

#### Flexor carpi radialis (FCR) muscle weight

Being innervated exclusively by the MN, this muscle’s weight was used to assess motor reinnervation in the territory of the MN. After euthanizing the rats as described above, the muscle was harvested on both sides from its origin until its distal tendon insertion. Both muscles were weighed using a precision scale, KERN770®, which had a precision of 0.1 mg.[46, 48]

#### Histological evaluation

The MN distally to the repair site and the middle portion of the nerve conduit used to bridge the defect were harvested after euthanasia. The specimens were fixed in 10% paraformaldehyde, and prepared for histological examination, using hematoxylin-eosin and Masson’s trichrome stains, as well as immunohistochemistry for neurofilaments, peripherin and acetylcholinesterase (**Table 1**).[75–78]

**Table 1:** Synthesis of the immunohistochemical methods used by the authors to stain the different types of nerve fibers.

Nerve fiber		Numerical classification of nerve fibers	Innervated structures / function	Nerve fiber diameter (μm)	Myelination	Conduction velocity (m/s)[79]
type	subtype					
**A**	**α**	**Ia**	Muscle spindle annulospiral receptor (main responsible for proprioception)	12–22	Thickly myelinated	70–120
			Extrafusal skeletal muscle fibers (voluntary motor control)			
**A**		**Ib**	Golgi tendon organ (contractile force)	12–22	Thickly myelinated	70–120
**A**	**β**	**II**	Pressure, touch and vibration receptors of the skin (cutaneous mechanoreceptors sensibility) Muscle spindle flower spray receptors (secondary responsible for proprioception)	5–12	Thickly myelinated	30–70
**A**	**γ**	**II**	Intrafusal skeletal muscle fibers (muscle tone control)	2–8	Thickly myelinated	15–30
**A**	**δ**	**III**	Some nociceptors (sharp pain), cold receptors, most hair receptors (touch and pressure), some visceral receptors	1–5	Thinly myelinated	5–30
**B**	**-**	**-**	Preganglionic autonomic efferents	< 3	Thinly myelinated	3–15
**C**	**-**	**IV**	Most nociceptors (dull, aching pain), warmth receptors, some mechanoreceptors, itch receptors, some visceral receptors, postganglionic autonomic efferents	0.1–1.3	Nonmyelinated	0.6–2.0

The authors used immunohistochemistry for neurofilaments (blue areas), acetylcholinesterase (red areas) and peripherin (brown areas).[50, 80–83] Neurofilament staining marks virtually all nerve fibers. Acetylcholinesterase staining highlights mostly motor nerve fibers.[84] Most sympathetic nerve fibers are marked by peripherin staining. Fibers that do not stain by acetylcholinesterase and peripherin are predominantly myelinated sensory fibers.[82] The combination of these immunohistochemical methods roughly allows to functionally dissect peripheral nerves.[75]

**Histomorphometric evaluation** was performed independently by two blinded observers, using the software Fiji®. In cases of discrepancies superior to 5%, the histological sections were reviewed by the two observers. The following parameters were determined in the MN section immediately distal to the repaired nerve gap: cross section area in a transverse section, total number of nerve fibers (neurofilament positive), number of acetylcholinesterase positive nerve fibers, number of peripherin positive nerve fibers, number of acetylcholinesterase–and peripherin negative fibers. Furthermore, the vascular density in the middle of the reconstructed segment was also determined. Vascular density inside the reconstructed median nerve segment was determined based on counts of the vessels inside the epineurium, as the vessels over the epineurium mixed with the surrounding tissues in an indistinct manner, making the establishment of boundaries between the epineurium and surrounding tissues frequently impossible. [75, 85]

The number of structures inside nerve segments was calculated by the product of the cross-section area of the respective nerve segment (assessed on 4X amplification) and of the density of the structure of interest. Density was determined by counting the average number of structures in 3 random 20X amplification fields and dividing this value by the area of that field. To avoid under or overestimation of structures number, structures included in counts only if the top upper edge of the structure was included in the microscopic field.[86, 87]

**Retrograde neuron marking and fluorescence microscopy evaluation**: On D90, after performing the functional examinations described above, and with the rat under anesthesia, 12 μl of 5% True Blue Diaceturate (TB, Sigma®) e 12 μl of 3% of Lucifer Yellow Dilithium salt (LY, Sigma®) were injected intradermally in the skin territory of the MN in the right hand (at the level of the radial hand pads) and in the right flexor carpi radialis muscle, respectively, using 27-gauge intradermic needles (BD Bioscience™).[41, 55, 88] This allowed to morphologically evaluate the sensory and motor reconstruction of the MN across the nerve gap.[41, 48, 89, 90]

On D100, rats were again anesthetized with a mixture of ketamine (5 mg/kg) and diazepam (0.25 mg/kg) given intraperitoneally.[43] Subsequently, a ventral thoracotomy was performed. Rats were euthanized by sectioning the cranial vena cava and exsanguination. After euthanizing the rat, the following structures were removed: MN proximal to the reconstructed MN, the C7 spinal cord segment in continuity with the dorsal and ventral C7 spinal nerve roots and including the C7 dorsal root ganglion (DRG) on both sides. A left parasagittal section was made in the ventral surface of the spinal cord segment, in order to convey information on laterality. These nerve structures were immersed in 4% paraformaldehyde and 10% sucrose in 0.1 M phosphate buffered saline (PBS) at pH 7.4 for 48 hours for fixation. After fixation, the specimens were transferred into increasing concentrations of sucrose in PBS for at least 15 hours for each concentration (15% and 30%). The specimens were then frozen in liquid nitrogen. Subsequently, transverse cryostat sections were cut at 20 μm for the DRG and the MN and at 50 μm for the spinal cord segments. These sections were then thaw-mounted on polylysine-coated glass slides.[91, 92]

Specimens were observed by epifluorescence under a fluorescence microscope.

The number of marked nerve fibers in the proximal aspect of the MN was assessed as described above. For each DRG, the number of True Blue labelled cells was semi quantitatively assessed by counting the fluorescent cells on what appears to be the largest cross section. For each C7 spinal cord, the region with greatest fluorescence in the ventral horn was searched with a 4X amplification. In this area, the average number of Lucifer Yellow stained cells was determined based on counts done in 3 random 20X-amplification fields.[41, 45]

### Statistical analysis

Qualitative variables were expressed as percentages. Quantitative variables were expressed as means ± SD. IBM SPSS Version 21.0® software was used for descriptive and inferential statistical analysis. The Kolmogorov-Smirnov test was used to assess whether variables were distributed normally. Analysis of variance with a Bonferroni *post hoc* test was used to compare averages in normally distributed data (rat weight, running ladder velocities, morphometric measurements in walking track evaluations, IRT, strength evaluation and ENMG measurements, FCR weight and histomorphometrical data). Kruskal-Wallis test was used to compare means in non-normally distributed data (grasping test). Proportions were analyzed with the chi-square test. Association between numerical variables was investigated using Pearson’s correlation coefficient. Relationship between ordinal variables was evaluated with resort to Spearman Rank Correlation Coefficient. Kaplan Meier survival analysis was performed to identify differences between groups regarding time to recovery of a positive grasping test.

A two-tail value of p<0.05 was considered to be statistically significant.

## Results

### Rat mortality was higher in the PNF group

The total number of rats reaching the end of the experiment was 87. These animals were distributed in the experimental groups as follows: 17 in the Sham group; 17 in the Excision group; 10 in the NG group; 20 in the CNF group; 15 in the ANVF group; and 8 in the PNF group (**Table 2**). All rats died in the perioperative period (<48 hours after surgery). Mortality rate was higher in the PNF group than in the remaining experimental groups (60% versus 21%; p<0.05). In the former group, 10 deaths occurred in the first 24 hours after the first surgery, and the remaining 2 deaths in the day after the second surgery. In all the deceased animals necropsy examination was performed by both the first author and by another author who is a certified pathologist (LML). Examination of all the rats in the PNF group that had deceased after the first surgery revealed a large hematoma in the groin region where the arterial-venous fistula had been created. In all the other prematurely deceased rats, a large hematoma was observed in the operated arm. No other abnormal findings were observed at autopsy. Hypovolemic shock was deemed to be the most likely cause of death in all the dead rats.

**Table 2:** Mortality, weight gain, grasping test, ladder running test and pin prick test results throughout the experiment.

Parameter		Sham group	Excision group	NG group	CNF group	ANVF group	PNF group	Relevant findings
**Mortality**		15%	15%	50%	0%	25%	60%	Mortality rate was higher in the NG and the PNF groups than in the remaining experimental groups (p<0.05)
**Animal weight gain**(%)		79.4 ± 4.9	74.2 ± 3.8	83.3 ± 6.2	74.0 ± 6.8	75.3 ± 7.6	79.9 ± 6.1	No significant differences
**Time to recovery of grasping (days)**		0 (immediately after surgery)	87.31 ± 4.41	75.00 ± 5.86	45.75 ± 2.98	34.00 ± 2.30	97.50 ± 1.53	Fastest recovery of grasping was observed in the CNF and ANVF groups (p<0.001)
**Average grasping strength**	**D30**	4.00 ± 0.00	0.00 ± 0.00	0.00 ± 0.00	0.60 ± 0.82	0.67 ± 0.62	0.00 ± 0.00	On D90, grasping strength was greater in the CFN and ANVF than in the Excision, Nerve graft and PNF groups
	**D45**	4.00 ± 0.00	0,06 ± 0.24	0.00 ± 0.00	1.10 ± 1.02	1.60 ± 0.51	0.00 ± 0.00	
	**D60**	4.00 ± 0.00	0.18 ± 0.39	0.60 ± 0.70	2.30 ± 1.13	2.60 ± 1.05	0.00 ± 0.00	
	**D75**	4.00 ± 0.00	0.29 ± 0.47	0.80 ± 0.63	3.00 ± 1.01	2,80 ± 1.01	0.00 ± 0.00	
	**D90**	4.00 ± 0.00	0.35 ± 0.49	1.30 ± 0.95	3.80 ± 0.41	3.80 ± 0.41	1.50 ± 1.20	
**Running Velocity in the ladder (cm/s)**	**D30**	57.17 ± 31.02	21.10 ± 6.17	15.29 ± 13.14	32.06 ± 13.30	31.81 ± 9.93	15.51 ± 6.24	On D90 there were no statistical significant differences between sham and the CNF, the ANVF and the PNF groups
	**D45**	58.74 ± 37.33	21.65 ± 6.57	15.32 ± 4.76	29.54 ± 7.22	34.12 ± 4.80	28.08 ± 9.85	
	**D60**	60.26 ± 39.96	21.56 ± 4.85	29.42 ± 12.97	40.96 ± 10.60	35.76 ± 8.62	39.66 ± 17.53	
	**D75**	64.63 ± 36.08	23.48 ± 4.86	30.91 ± 22.98	43.13 ± 8.36	48.88 ± 11.61	35.06 ± 7.23	
	**D90**	63.34 ± 31.58	23.30 ± 5.05	39.83 ± 14.33	54.46 ± 19.13	56.71 ± 10.21	39.23 ± 14.05	
**Pin Prick test**(%)	**D30**	97.05 ± 4.70	5.88 ± 9.39	21.71 ± 12.10	39.18 ± 44.54	75.06 ± 18.74	2.50 ± 4.63	On D90, the best results were observed in the Sham, CNF and the ANVF groups (p<0.001)
	**D45**	107.67 ± 9.12	18.69 ± 21.89	19.11 ± 16.82	69.86 ± 27.93	82.17 ± 13.80	24.17 ± 22.51	
	**D60**	102.21 ± 4.91	13.33 ± 4.46	20.44 ± 6.73	93.89 ± 20.12	83.33 ± 14.80	35.07 ± 3.17	
	**D75**	93.33 ± 5.09	16.74 ± 7.90	40.00 ± 29.06	93.00 ± 4.70	94.01 ± 8.28	37.57 ± 27.04	
	**D90**	100.20 ± 6.47	7.84 ± 10.95	42.00 ± 18.14	87.24 ± 17.63	107.03 ± 29.20	48.21 ± 29.81	

**NG**, nerve graft; **CNF**, conventional nerve flap; **ANVF**, arterialized neurovenous flap; **PNF**, prefabricated nerve flap

**D**, day after the beginning of the experiment

**Average grasping strength** was evaluated semi quantitatively using a scale of 0 to 4 (0, no grasping; 1, flexes fingers only without opposition; 2, flexes fingers against minimal opposition; 3, flexes fingers against opposition but with less strength than the contralateral limb; 4, flexes fingers with the same strength as the contralateral limb.

Pin prick test results are expressed as percentages of the average contralateral values.

Numeric variables are expressed as average ± standard deviation.

### Daily observation of rats did not reveal signs of distress

General health and behavior of the experimental animals was adequate throughout the experiment. All rats presented moderate to high levels of activity, grooming themselves regularly. None of the animals presented autophagy or self-mutilation. The surgical wounds healed uneventfully. Skin ulcers were not observed on the operated paws.

### Animal weight gain did not vary significantly among the experimental groups

At the end of the experiment, the average weight gain was 79.4% ± 4.9% for the Sham group; 74.2% ± 3.8% for the Excision group; 83.3% ± 6.2% for the NG group; 74.0% ± 6.8% for the CNF group; 75.3% ± 7.6% for the ANVF group; and 79.9% ± 6.1% for the PNF group. These differences were not statistically significant (**Table 2**).

### The grasping test revealed faster and more complete motor recovery in the CNF and in the ANVF groups than in the NG group

**Fig 5:**
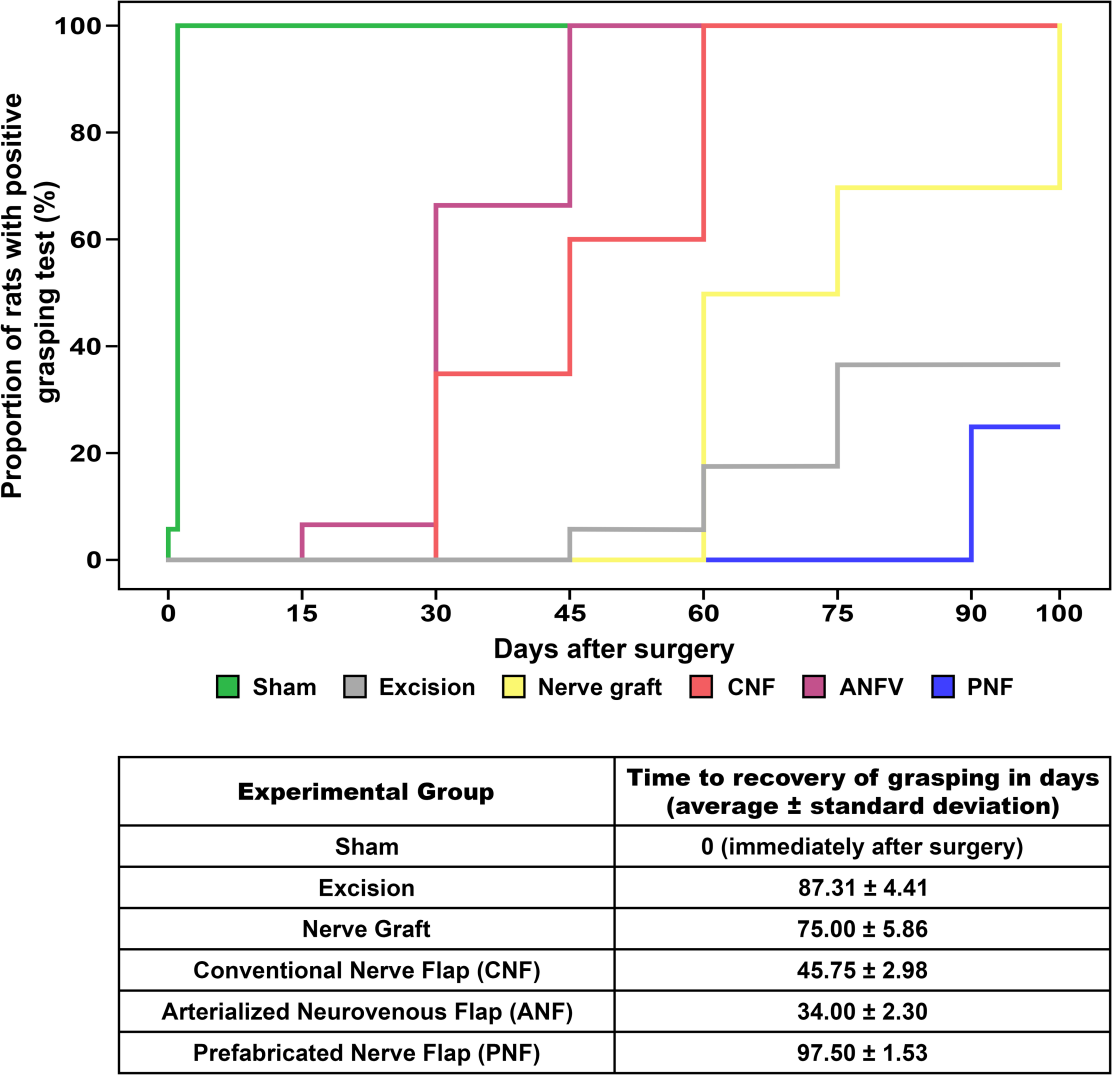
Time to recovery of grasping in the operated limb. Fastest recovery of grasping was observed in the CNF and ANVF groups (p<0.001).

**Fig 6:**
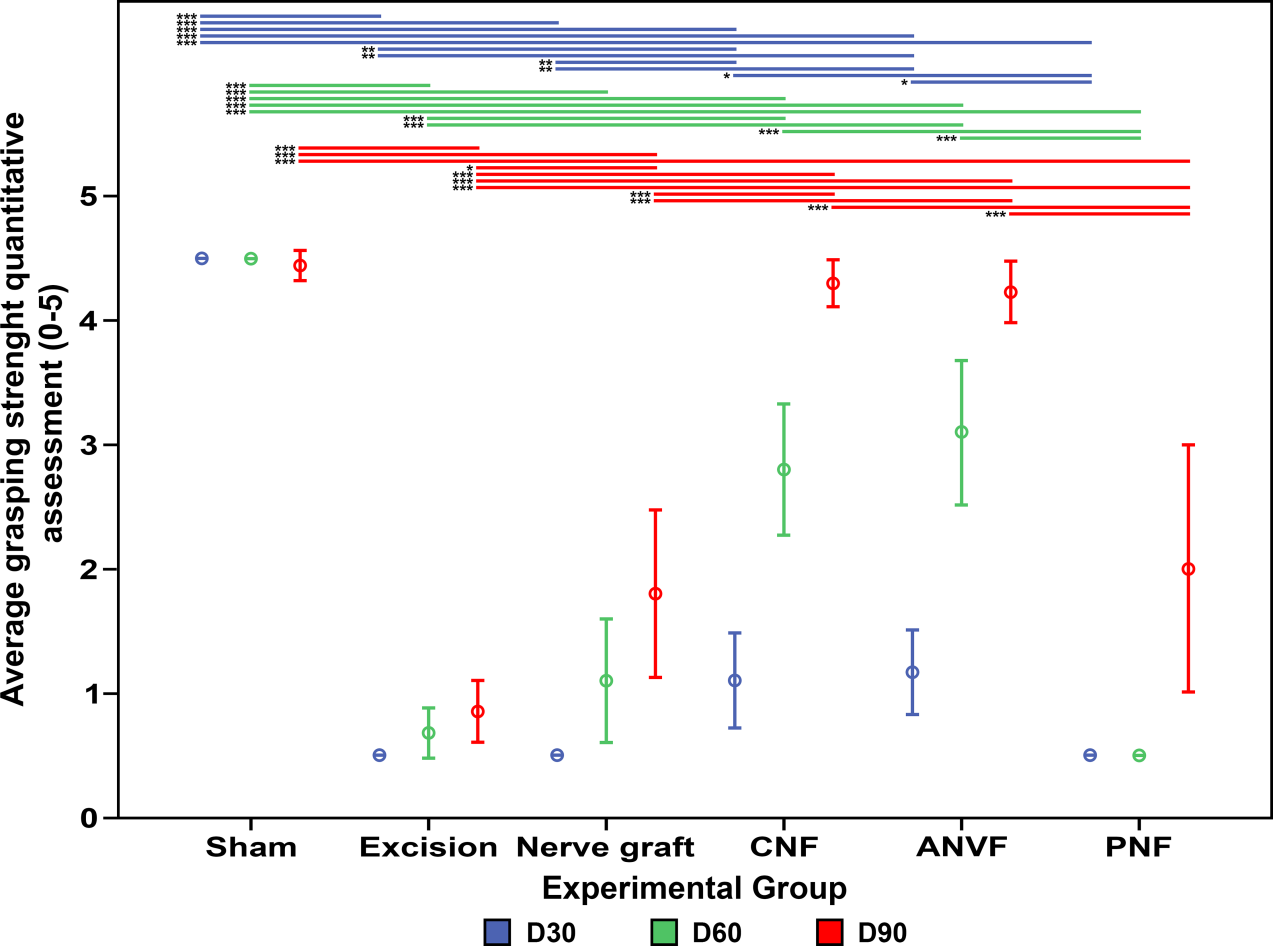
Qualitative assessment of grasping strength in the operated limb in the different experimental groups 30, 60 and 90 days after the reconstruction of the median nerve gap. Vertical bars represent 95% confidence intervals. Horizontal lines in the upper portion of the figure indicate statistically significant differences between groups (p<0.05). *, p<0.05; **, p<0.01; ***, p<0.001.

Recovery of grasping occurred in the immediate postoperative period in all rats in the Sham Group. In all other groups, no grasping was observed immediately after surgery (**Fig 5**). Fastest recovery of grasping was observed in the CNF and ANVF groups (p<0.001). At the end of the experiment, grasping strength was greater in the CFN and ANVF than in the Excision, Nerve graft and PNF groups (**Fig 6**). In fact, on D90, there were no statistically significant differences between the CNF, ANVF and the Sham groups (**Fig 6**).

### Running ladder test revealed comparable velocities at the end of the experiment between the Sham group and the CNF, the ANVF, and the PNF

**Fig 7:**
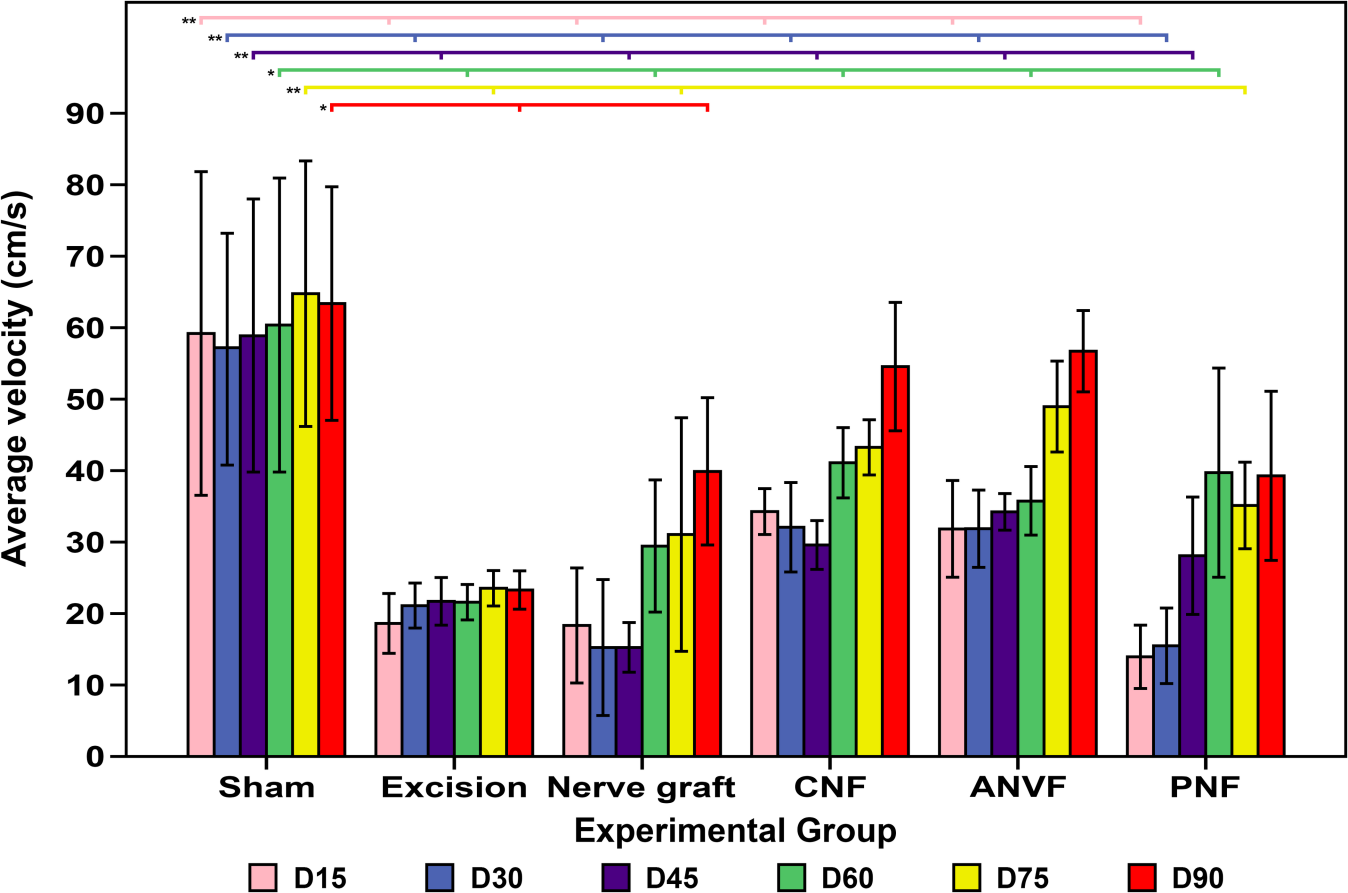
Average velocity in the ladder running test in the different experimental groups during the experiment. Vertical bars represent 95% confidence intervals. Horizontal lines in the upper portion of the figure indicate statistically significant differences between groups on the 90^th^ day postoperatively (p<0.05). *, p<0.05; **, p<0.01; ***, p<0.001.

On D15, the average velocity in the running ladder test was greater in the Sham group than in all other groups (p<0.01; **Fig 7**; **Table 2**). The superiority of this group was maintained until D60. From this time on, there was no statistical difference between this group and the CNF and PNF groups. On D90 there were no statistical significant differences between sham and the CNF, the ANVF and the PNF groups. At the end of the experiment, the CNF and ANVF groups presented better average velocities than the NG group but these differences were not statistically significant (**Fig 7**).

### Pin prick test revealed better nociceptive recovery in the CNF and ANVF groups

**Fig 8:**
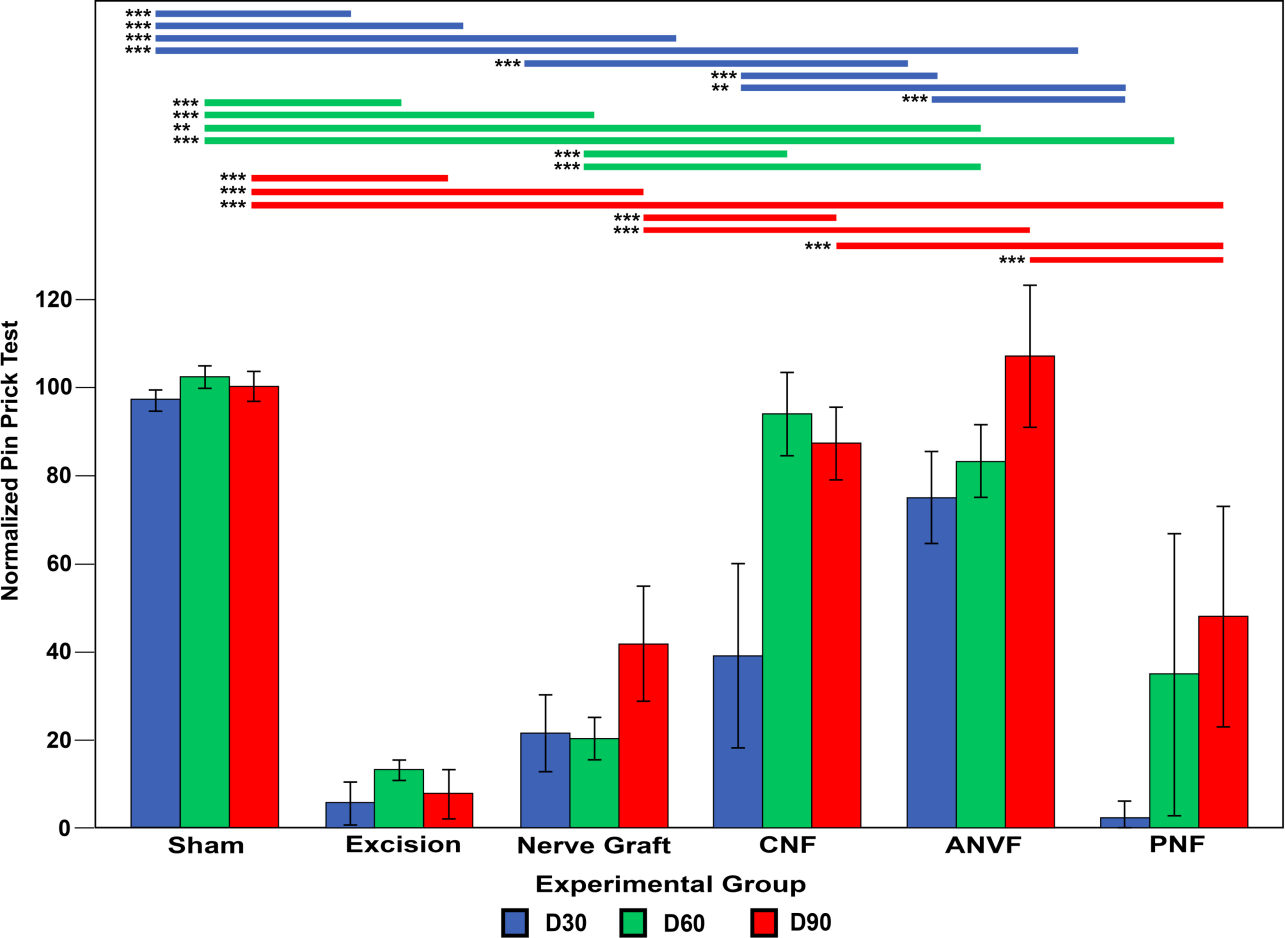
Nociceptive evaluation using cumulative pin prick test results in the operated forelimb normalized to the contralateral limb in the different experimental groups throughout the experiment. Vertical bars represent 95% confidence intervals. Horizontal lines in the upper portion of the figure indicate statistically significant differences between experimental groups (p<0.05). **, p<0.01; ***, p<0.001.

Thirty days after surgery, the ANVF group presented the best average sensory recovery in the Pin Prick test, showing no statistical significant difference relatively to the Sham group (**Fig 8**; **Table 2**). On D60 and D90, the best results were observed in the Sham, CNF and the ANVF groups. The latter two groups presented average scores significantly superior to those observed in the NG and PFN groups (p<0.001; **Fig 8**).

### Walking track analysis revealed that the rate of radial deviation was lower in groups in which a vascularized nerve conduit was used

**Fig 9:**
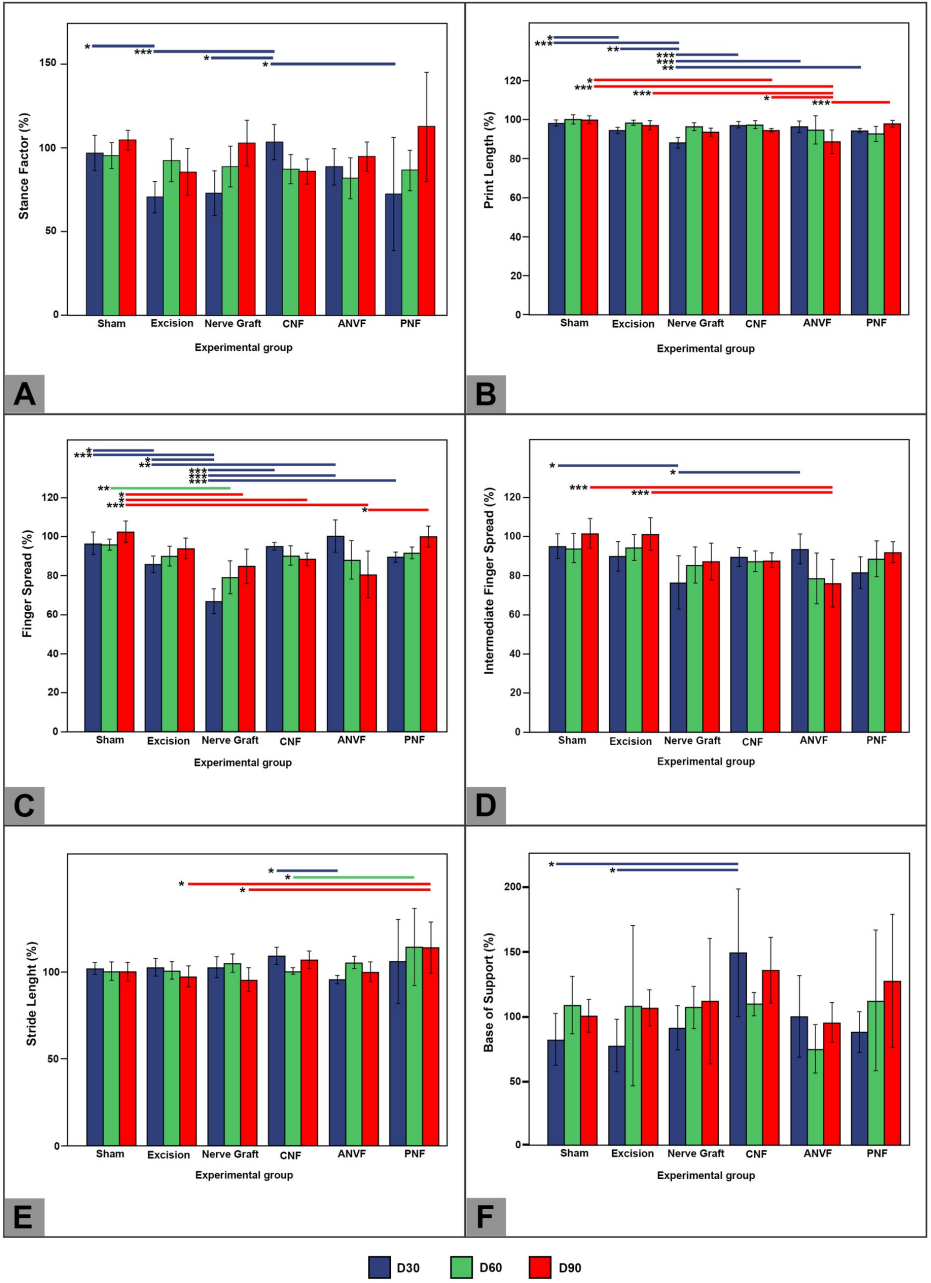
Walking track analysis of the right forelimb (operated paw) of rats in the different experimental groups throughout the experience. Values are expressed as percentages of averages normalized to the contralateral side. (A) Stance factor. (B) Print length; (C) Finger spread factor; (D) Intermediate finger spread factor; (E) Stride length; (F) Base of support. Vertical bars represent 95% confidence intervals. Horizontal lines in the upper portion of the figure indicate statistically significant differences between experimental groups (p<0.05). *, p<0.05; **, p<0,01; ***, p<0.001.

On D30 the CNF group presented a better normalized stance factor than the excision, NG and PNF groups (p<0.001; **Fig 9A**; **S1 Table**). Subsequently, no statistically significant differences were found between groups. The average normalized print length in the operated limb was higher in the CNF, ANVF and PNF than in the NG on D30. On D60 no differences were found between groups. On D90, CNF and PNF presented better results than the ANVF group regarding the normalized print length (**Fig 9B**). Pertaining to normalized finger spread and intermediate finger spread, on D30, better results were observed in the groups using vascularized conduits compared to the NG group (p<0.05; **Fig 9C** and **9D**). However, at the end of the experiment these differences were no longer visible. Finally, stride analysis failed to reveal meaningful differences between the different groups (**Fig 9E** and **9F**).

**Fig 10:**
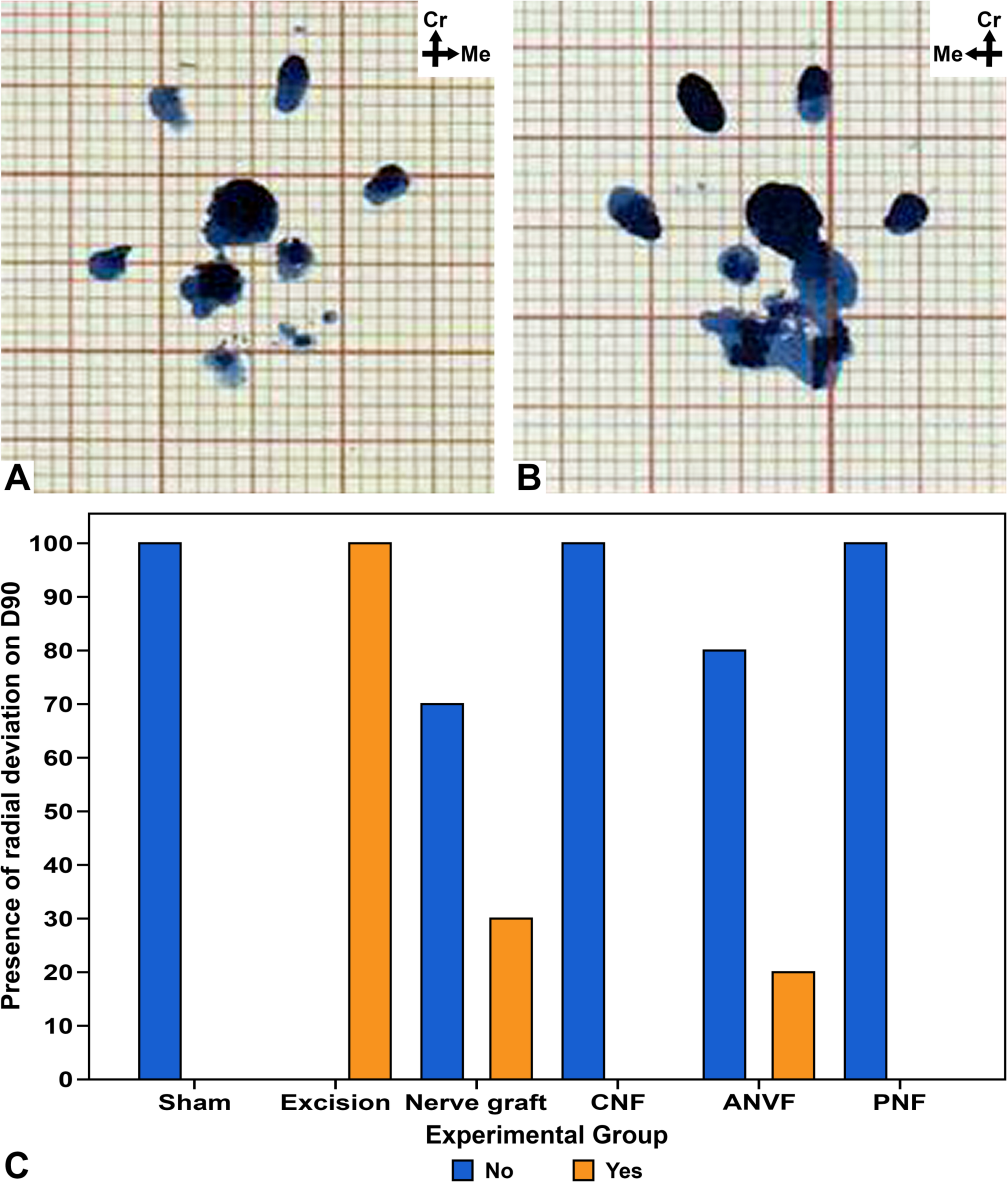
Presence of radial deviation in the walking tracks of the operated forepaws in the different experimental groups at the end of the experiment. (A) Left forepaw print of a rat in the nerve graft group, showing a normal impression. (B) Right forepaw print of the same rat, showing radial deviation of the paw. Radial deviation was defined by radial (medial) rotation of first four digits, as well an angle between the middle point of the most caudal aspect of the hand and the middle point of the most cranial aspect of the second and fourth digits that was at least 10° smaller comparatively to the contralateral side. (C) Bar graph showing the proportion of rats with radial deviation of the operated forepaws at the end of the experiment.

The authors observed that some rats presented in the forepaw impressions of the operated side a radial (medial) rotation of first four digits, as well an angle between the middle point of the most caudal aspect of the hand and the middle point of the most cranial aspect of the second and fourth digits that was at least 10° smaller comparatively to the contralateral side. The author defined this morphological pattern as **radial deviation of the forepaw**. This pattern was found in the operated forepaw print on D30 in 94.1% of the rats in the Excision group, in 100% of the rats in the NG group, in 40% of those in the CNF group, and in 13.3% in ANVF (**Fig 10A** and **10B**). On D60, 100% of the rats in the Excision and the NG groups presented radial deviation, while only 20% of the rats in the ANVF group presented radial deviation. From D60 on, no radial deviation was observed in the CNF group. On D90, radial deviation was observed in 100% of the Excision group, 30% of the NG group, and 20% of the ANVF group (**Fig 10C**). Radial deviation was never observed in the animals in the Sham and in the PNF groups. For each assessment day, the rate of radial deviation was lower in the groups in which a vascularized nerve conduit was used compared to the NG group (p<0.001).

### Thermographic assessment failed to show differences between groups

**Fig 11:**
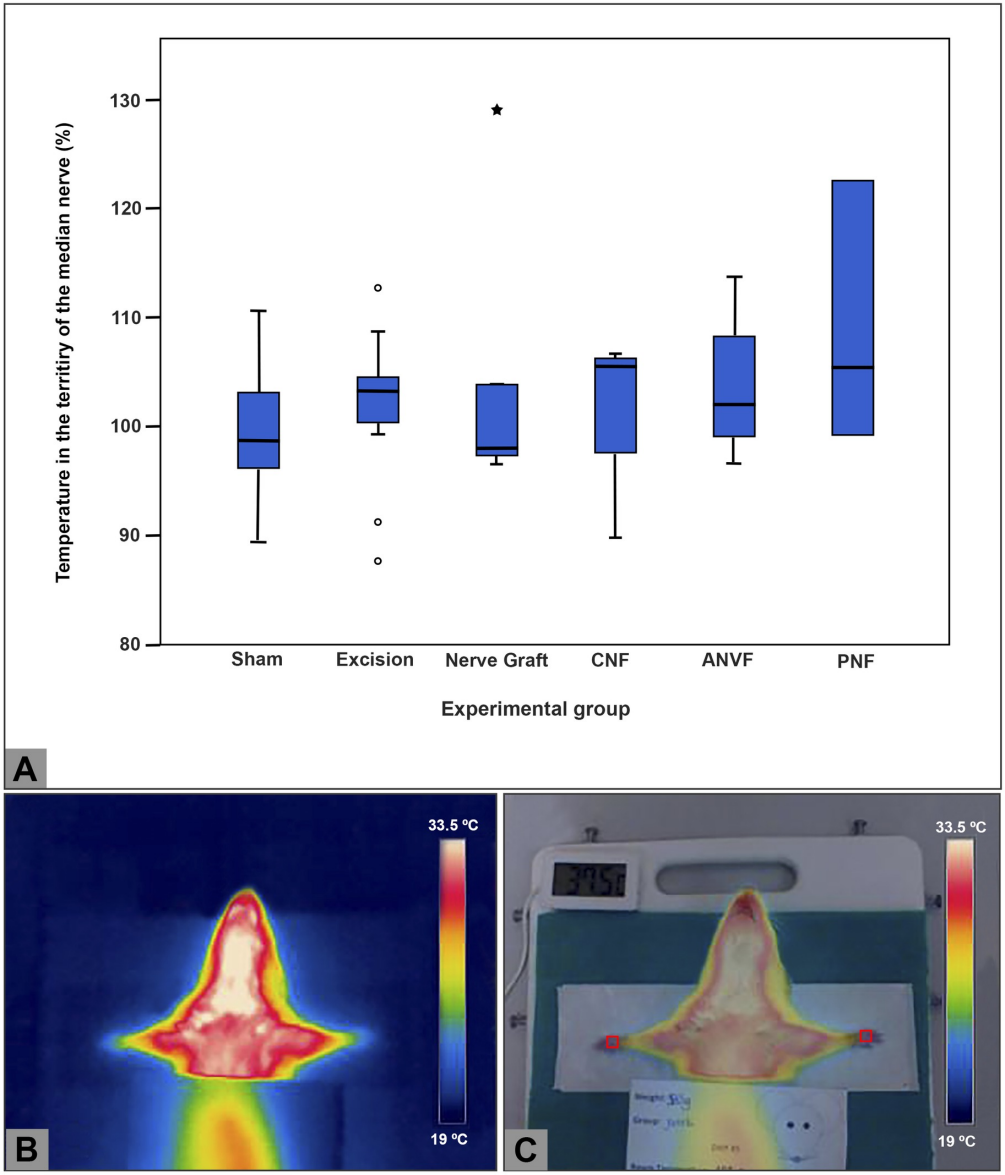
Temperature on the surface of the skin territory of the median nerve. (A) Boxplot graphic illustrating the average temperature in the skin territory of the right median nerve relatively to that of the contralateral side. Temperature measurements were made using infra-red thermography. (B) Typical thermography image. (C) Image resulting from the overlap of the thermography image and of the digital photographic image. This allows to evaluate the temperature in the territory of the median nerve.

IRT of the skin of the MN territory in the right forepaw revealed an average normalized temperature of 99.4% ± 5.9%; 101.7% ± 7.1%; 104.9% ± 13.7%; 101.8% ± 8.0%; 103.6% ± 7.2%; and 109.0% ± 10.8% in the Sham, Excision, NG, CNF, and PNF groups, respectively, compared to the contralateral side (**Fig 11; S2 Table**). These differences were not statistically significant. Analogously, no differences were found pertaining to the maximal and minimal temperatures on the surface of the forepaws (**S2 Table**).

### Electroneuromyographic assessment revealed a lower motor stimulation threshold in the CNF and ANVF than in the NG group

**Fig 12:**
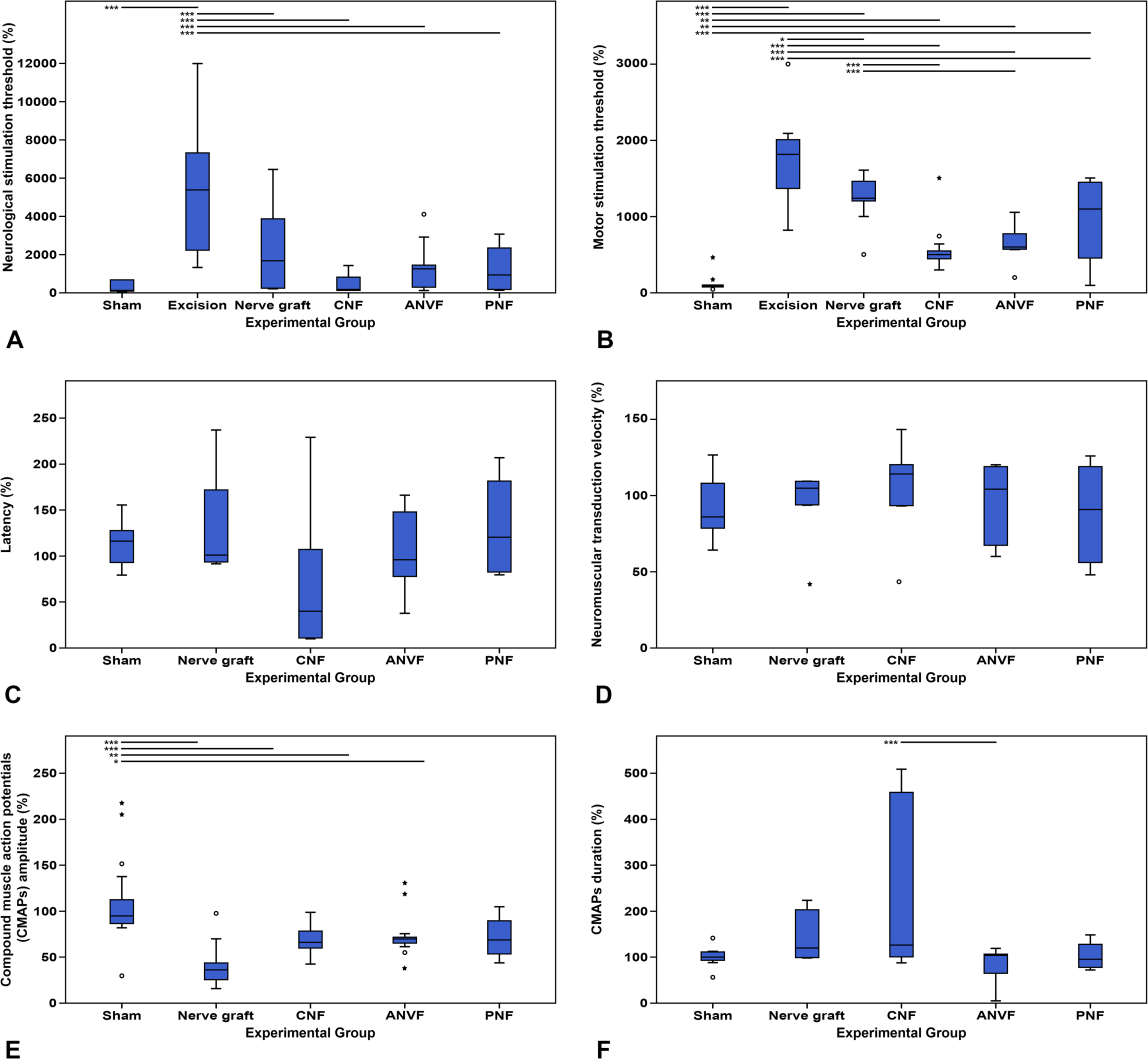
Electroneuromyographic assessment of the right forelimb (operated paw) of rats in the different experimental groups throughout the experience. Values are expressed as percentages of averages normalized to the homologous contralateral side average values. (A) Neurological stimulation threshold; (B) Motor stimulation threshold; (C) Latency; (D) Neuromuscular transduction velocity; (E) Compound muscle action potentials (CMAPs) amplitude; (F) CMAPs duration. Vertical lines represent 95% confidence intervals. Horizontal lines in the upper portion of the figure indicate statistically significant differences between experimental groups (p<0.05). *, p<0.05; **, p<0,01; ***, p<0.001.

**Fig 13:**
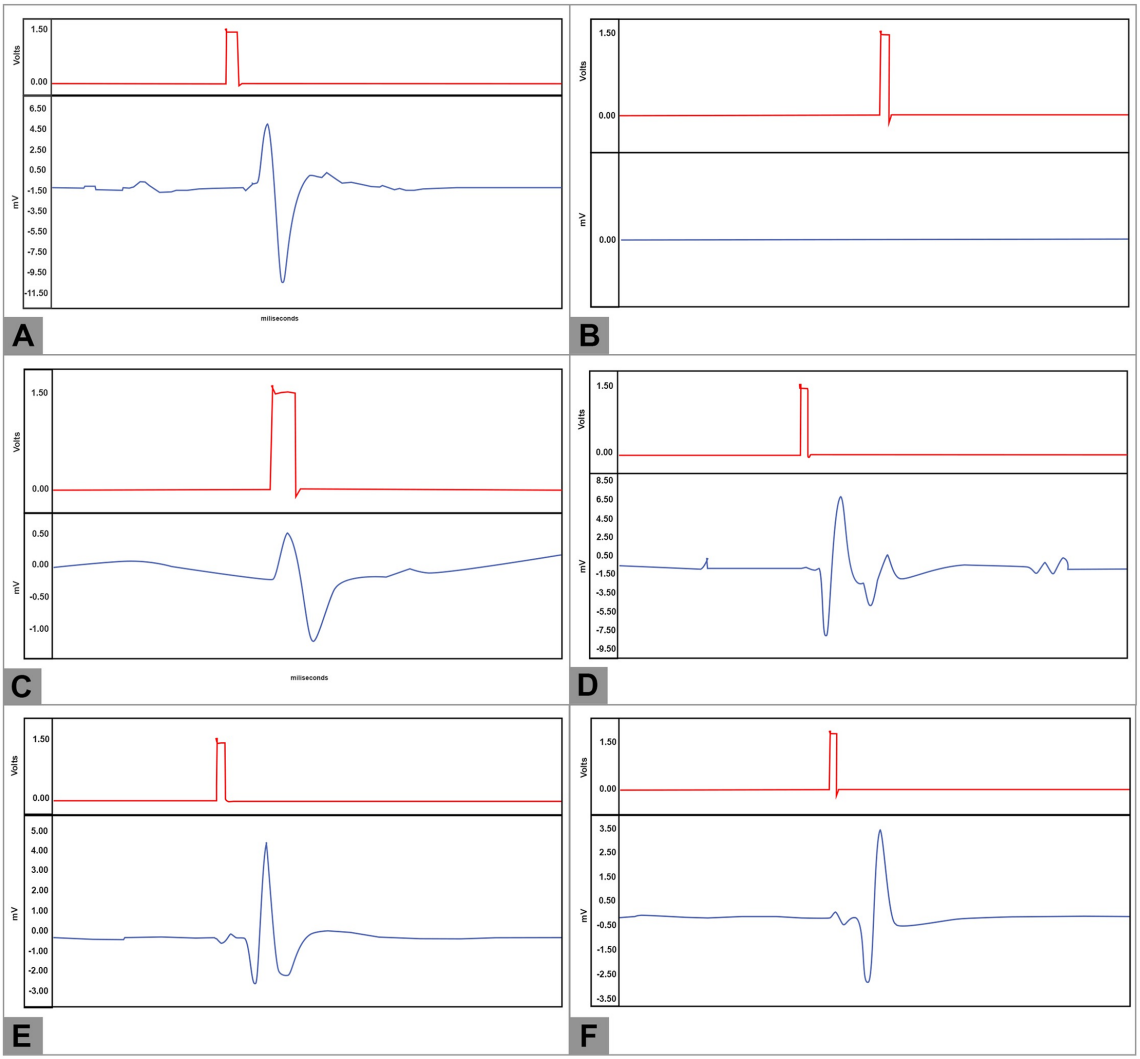
Typical compound muscle action potentials patterns in the different experimental groups. (A) Sham group and left paw of the rats in the other experimental groups. (B) Excision group. (C) Conventional nerve flap group. (D) Arterialized neurovenous flap group. (E) Prefabricated nerve flap group.

On D90, the normalized neurological stimulation threshold was significantly higher in the Excision group than in the remaining groups (p<0.001; **Fig 12A; Table 3**). In fact, no reproducible CAMP was obtained in the former group. No other differences were found relatively to normalized neurological stimulation threshold in the other groups. However, regarding the normalized motor stimulation threshold, the lowest values were obtained in the Sham group (p<0.01; **Fig 12B**). Lower values were obtained in the CNF and the ANVF groups than in the NG group (p<0.001; **Fig 12B**). No statistically significant differences were found in the latency and neuromuscular transduction parameters between the Sham group and the groups submitted to nerve gap reconstruction (**Fig 12C** and **12D**). Pertaining to the CMAP amplitude, higher values were obtained in the Sham group (110.63% ± 45.66%) than in the other groups submitted to nerve gap reconstruction (p<0.05; **Fig 12E**). These values were higher in the CNF group (69.53% ± 13.80%), in the ANVF group (73.34% ±22.86%) and the PNF group (71.68% ± 23.56%) than in the NG group (41.60% ± 24.4%). However, these differences did not meet statistical significance. Apart from a longer CMAP duration in the CNF group compared to the ANVF (p<0.001), no other significant differences were found between groups (**Fig 12F**). **Fig 13** shows the typical morphology of the CMAPs in the different experimental groups. The ENMG pattern in the NG group tended to be similar to that of the Sham group or to that of the contralateral non-operated side of all rats, apart from having a smaller amplitude (**Fig 13A** and **13B**). The ENMG pattern in the CNF, ANVF and in the PNF groups was invariably polyphasic, and showed a tendency to be of a longer duration but of a slightly lesser amplitude compared to the non-operated side (**Fig 13C** to **13E**). In the Excision group, no CMAPs were observed after applying an electrical stimulus to the MN.

**Table 3:** Electroneuromyographic assessment at the end of the experiment.

Parameter	Sham group	Excision group	NG group	CNF group	ANVF group	PNF group	Relevant findings
**Neurological stimulation threshold(%)**	281.63 ± 271.65	5359.98 ± 3466.52	2108.12 ± 2115.13	428.45 ± 472.87	1063.00 ± 807.61	1270.30 ± 482.72	On D90, this parameter was significantly higher in the Excision group than in the remaining groups (p<0.001)
**Motor stimulation threshold(%)**	462.52 ± 118.91	1694.10 ± 503.24	1249.50 ± 503.24	535.38 ± 253.15	619.46 ± 264.36	948.57 ± 592.41	Lower values were obtained in the CNF and the ANVF groups than in the NG group (p<0.001)
**Latency(%)**	113.55 ± 25.04	N/A	132.80 ± 69.95	72.82 ± 84.87	105.28 ± 52.41	131.97 ± 56.46	No significant differences between experimental groups
**Neuromuscular transduction velocity(%)**	92.01 ± 20.88	N/A	91.30 ± 26.51	100.06 ± 31.26	94.05 ± 26.33	88.15 ± 34.77	No significant differences between experimental groups
**CMAPs amplitude(%)**	110.63 ±45.66	N/A	41.60 ± 24.84	69.53 ± 13.80	73.34 ± 22.86	71.68 ± 23.56	No significant differences between experimental groups
**CMAPs duration(%)**	101.12 ± 23.92	N/A	151.06 ± 54.52	242.17 ± 185.97	82.87 ± 36.69	103.13 ± 31.24	Longer CMAP duration in the CNF group compared to the ANVF group (p<0.001)

**NG**, nerve graft; **CNF**, conventional nerve flap; **ANVF**, arterialized neurovenous flap; **PNF**, prefabricated nerve flap

**CMAP**s, compound muscle action potential.

**N/A**, non-applicable

All parameters are expressed as percentages of the average contralateral values.

Numeric variables are expressed as average ± standard deviation.

### Muscle strength was inferior in the NG group, although the differences did not meet statistically significance

**Fig 14:**
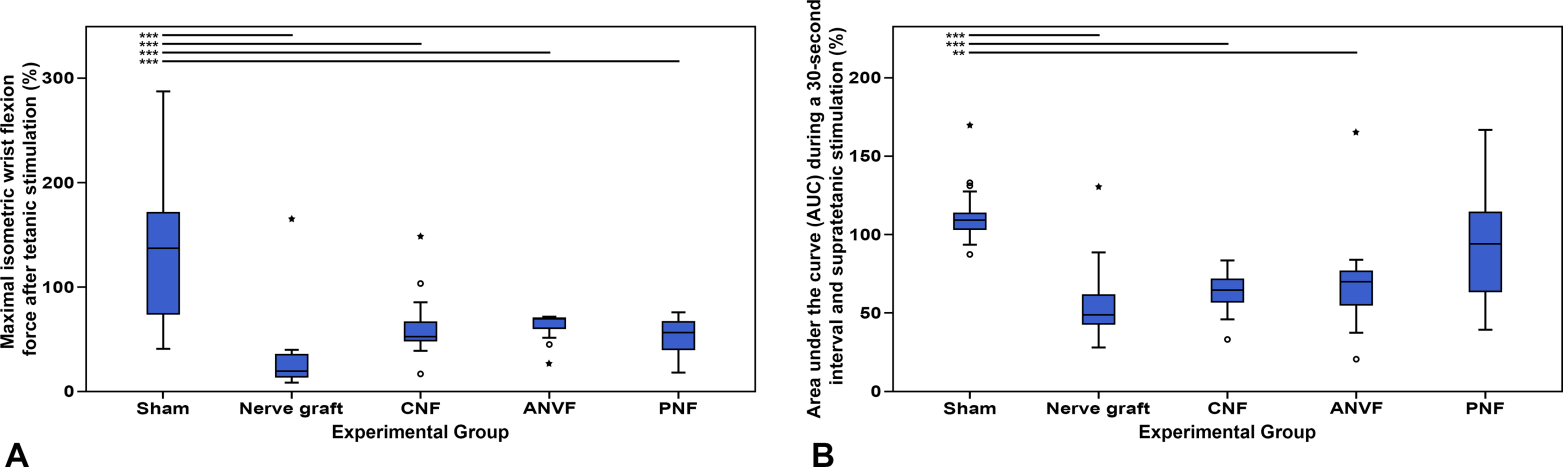
Muscle strength evaluation at the end of the experiment in the operated forelimb in the different experimental groups. (A) Maximal isometric wrist flexion force after tetanic stimulation. (B) Area under the curve (AUC) during a 30-second interval and supratetanic stimulation. Values are expressed as percentages of averages normalized to the homologous contralateral side average values. Vertical lines represent 95% confidence intervals. Horizontal lines in the upper portion of the figure indicate statistically significant differences between experimental groups (p<0.05). **, p<0,01; ***, p<0.001.

Normalized maximum isometric tetanic wrist flexion on the operated limb on D90 was 141.00% ± 75.55% in the Sham group, 35.67% ± 46.51% in the NG, 60.56% ± 27.59% in the CNF group, 63.05% ± 12.95% in the ANVF group, and 52.61% ± 18.73% in the PNF group. This value was significantly higher in the Sham group than in the other groups (p<0.001; **Fig 14A**). The normalized area under the curve during a 30-second interval and supra-tetanic stimulation was 112.54% ± 19.43% in the Sham group, 58.81% ± 29.63% in the NG group, 63.12% ± 12.43% in the CNF, 69.80% ± 31.67% in the ANVF group, and 93.61% ± 34.91% in the PNF group. Once again, the Sham group presented a better result in this parameter than all the other experimental groups, except for the PNF group (p<0.001; **Fig 14B**).

### Muscle weight was inferior in the NG and in the PNF groups

**Fig 15:**
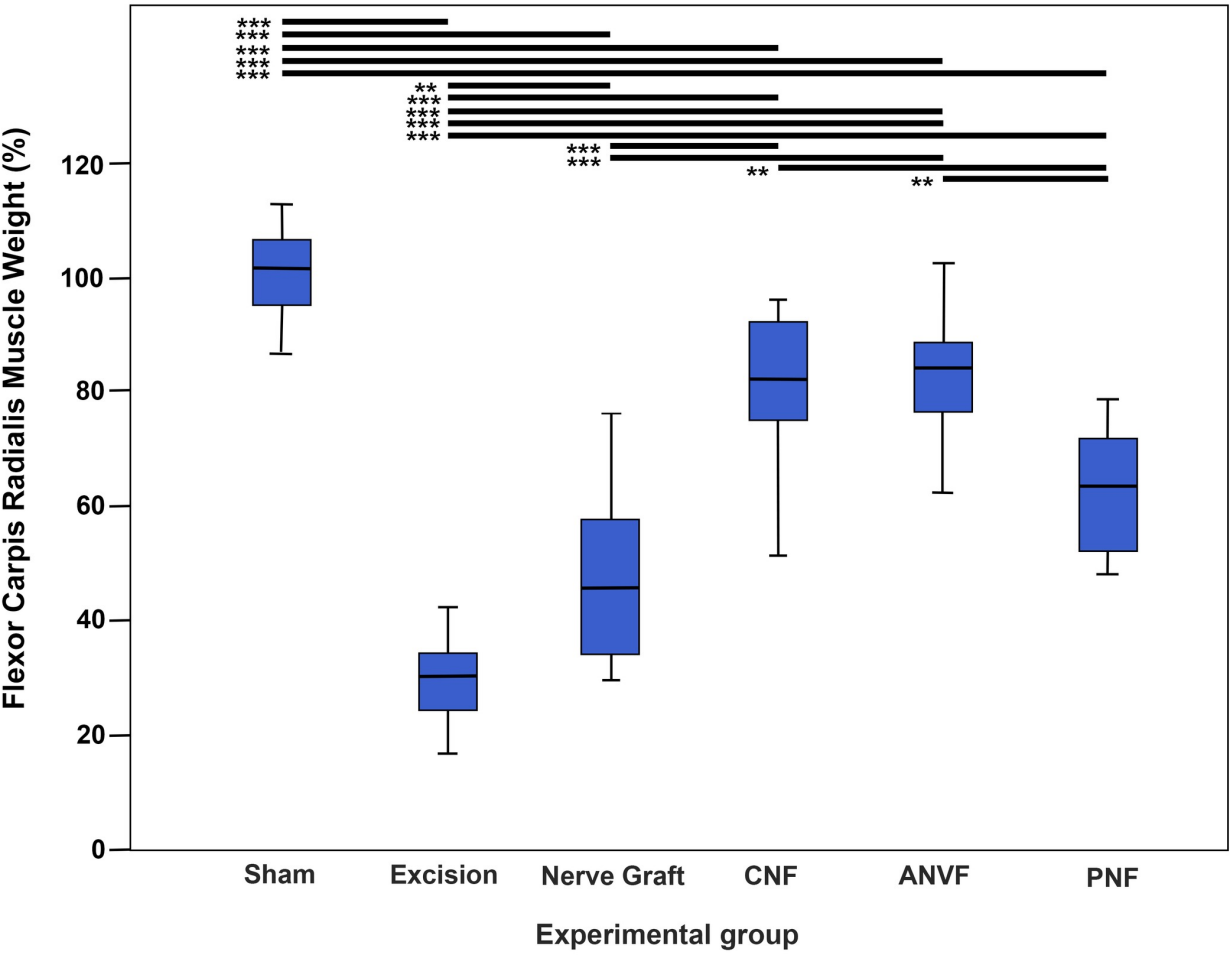
Flexor carpi radialis muscle weight of the right forelimb (operated paw) of rats in the different experimental groups. Values are expressed as percentages of averages normalized to the homologous contralateral side average values. Vertical lines represent 95% confidence intervals. Horizontal lines in the upper portion of the figure indicate statistically significant differences between experimental groups (p<0.05). p<0,01; ***, p<0.001.

**Fig 16:**
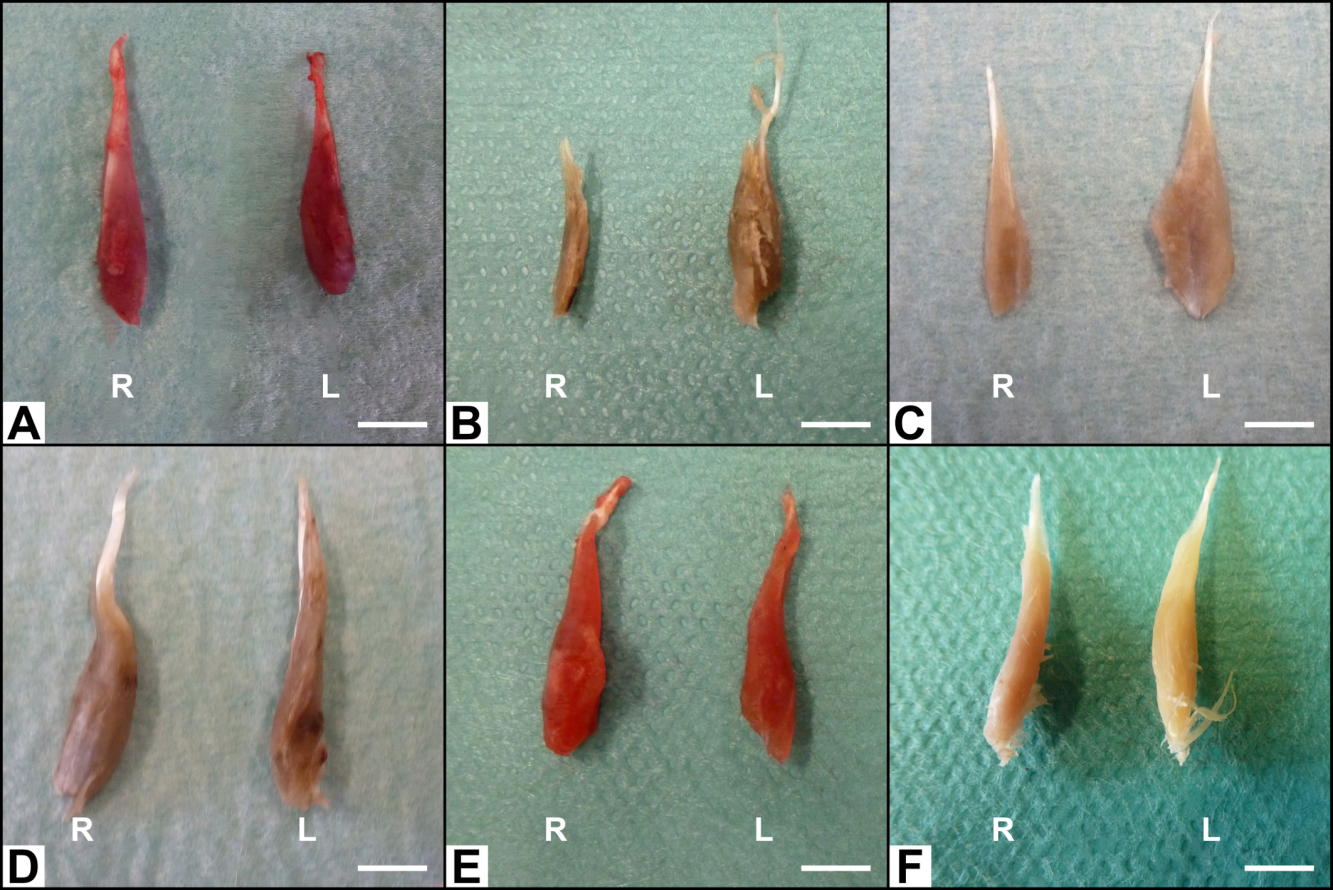
Photographs of the flexor carpi radialis muscle illustrating muscle gross appearance in the different experimental groups on the operated side (R, right) and on the non-operated side (L, left). (A) Excision group. (B) Nerve graft group. (C) Conventional nerve flap group. (D) Arterialized neurovenous flap group. (E) Prefabricated nerve flap group.

The normalized results for FCR muscle weight at the end of the experiment were 101.15% ± 8.14% for the Sham group, 30.24% ± 7.23% for the Excision group, 47.14% ±14.72 for the NG group, 80.29 ± 14.29% for the CNF group, 82.24% ±10.64% for the ANVF group, and 62.71% ± 11.12% for the PNF group (**Figs 15** and **16**). The Sham group presented a higher muscle weight than any of the other experimental groups (p<0.001). Among the groups using nerve conduits the CNF and the ANVF presented a better muscle weight than the NG and the PNF (p<0.01). The difference between these latter two groups was not statistically significant, although the PNF presented a higher average value.

### Histomorphometrical evaluation of the distal aspect of the median nerve showed a tendency to worse results in the NG group

**Fig 17:**
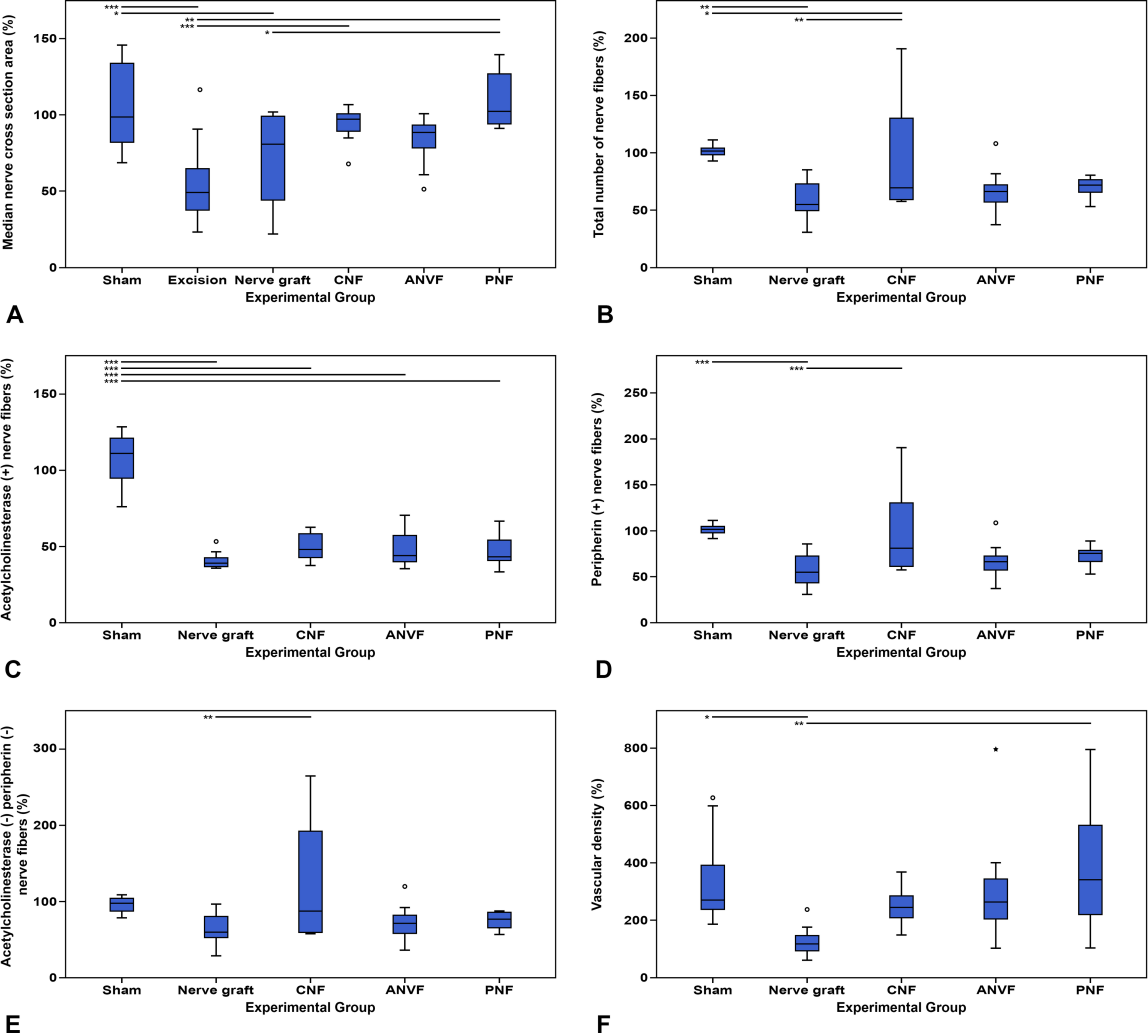
Histomorphometric evaluation of the right median nerve distally to the repair zone in the different experimental groups. Results are expressed as a percentage of the normal, contralateral side and are given as the mean. (A) Median nerve cross section area distally to the repair zone. (B) Total number of fibers (stained for neurofilaments) distally to the repair zone. (C) Acetylcholinesterase positive (+) nerve fibers distally to the repair zone. (D) Peripherin positive (+) nerve fibers distally to the repair zone. (E) Acetylcholinesterase negative (-) and peripherin negative (-) nerve fibers distally to the repair zone. (F) Vascular density in a cross section of the middle portion of the reconstructed nerve defect. Vertical lines represent 95% confidence intervals. Horizontal lines in the upper portion of the figure indicate statistically significant differences between experimental groups (p<0.05). *, p<0.05; **, p<0,01; ***, p<0.001.

**Fig 18:**
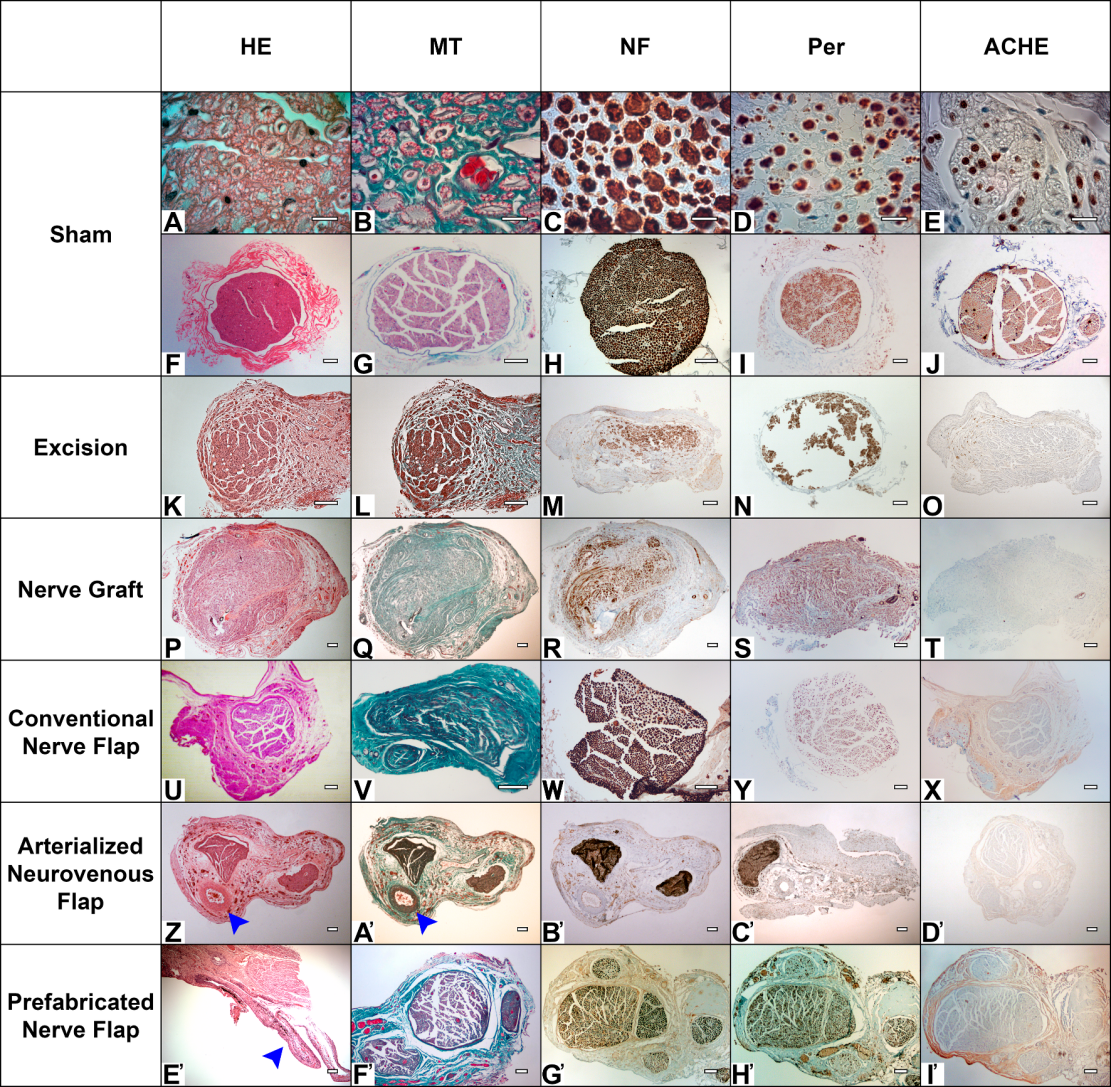
Representative histological features of the different experimental groups. HE, hematoxylin-eosin staining; MT, Masson’s trichrome staining; NF, neurofilament immunohistochemical staining; Per, peripherin immunohistochemical staining; ACHE, acetylcholinesterase immunohistochemical staining. Calibration bar (A to E) = 10 μm Calibration bar (F to I’) = 100 μm.

Histological examination of the median nerve distally to the reconstructed nerve segment revealed an inferior average cross section area in the Excision group (**Figs 17A** and **18**; **S3 Table**). Among the groups using nerve conduits, the NG presented an inferior area. However, this difference was statistically significant only between the NG and the PNF groups (p<0.05).

Regarding the internal structure of the distal portion of the MN, the total number of nerve fibers was significantly higher in the CNF than in the NG (p<0.01; **Fig 17B**). No other differences were found between the groups using conduits. The number of acetylcholinesterase positive fibers was higher in the Sham control group than in the remaining experimental groups (p<0.001; **Fig 17C**). Concerning this type of fibers, no differences were found in the latter groups. The average number of peripherin positive fibers was higher in the CNF and in the Sham groups than in the NG group (p<0.001; **Fig 17D**). Finally, the average number of acetylcholinesterase negative and peripherin negative fibers was once again higher in the CNF group than in the NG group (p<0.001; **Fig 17E**). Vascular density in the reconstructed nerve segment was lower in the NG group than in either the Sham or the PNF groups (p<0.05; **Fig 17F**). No statistically significant differences were found between the CNF, ANVF and the PNF groups.

### Histological characterization of the nerve conduits revealed greater architectural disorganization in the nerve graft conduit

Histological examination of the nerve conduits used to bridge the MN defect revealed preservation of the normal nerve architecture in the Sham group (**Fig 18A** to **18J**). In the Excision group, the distal stump of the MN showed clear signs of Wallerian degeneration in all the rats, as well as a proximal stump neuroma (**Fig 18K** to **18T**). In all rats in the NG group, there was a significant degree of fibrosis among the reconstituted nerve fibers. These fibrous septa divided the nerve structure in irregular bundles (**Fig 18P** to **18T**). In the CNF, the ANVF and the PNF groups, the reconstructed segment presented a single nerve fascicle (CNF and ANVF groups) or two nerve fascicles (PNF) whose fibers were disposed in a cohesive fashion (**Fig 18T** to **18I’**). In all the histological specimens in the ANVF group the brachial vein showed clear signs of arterialization. In addition, large tortuous veins could be seen in the proximity of the nerve segment (**Fig 18Z** and **18A’**). No clear signs of thrombosis were observed in the vein supplying flap in the ANVF group. In the histological sections of the PNF group it was also possible to observe arterialization of the venous fistula used to recruit the sciatic nerve segment (**Fig 18E’**). There was a greater density of large sized arterioles and venules in the epineurial region of these nerve segments than in any of the nerve conduits used in the other groups (**Fig 18F’**). Analogously, there is no evidence of vascular thrombosis of the vascular construct used to mobilize the nerve conduit. The primitive internal structure of the sciatic nerve was preserved until the most distal aspect of the nerve conduit in the PNF group (**Fig 18G’** to **18I’**).

### Retrograde axonal tracing using fluorescent markers showed anatomical restoration in all the groups using conduits

**Fig 19:**
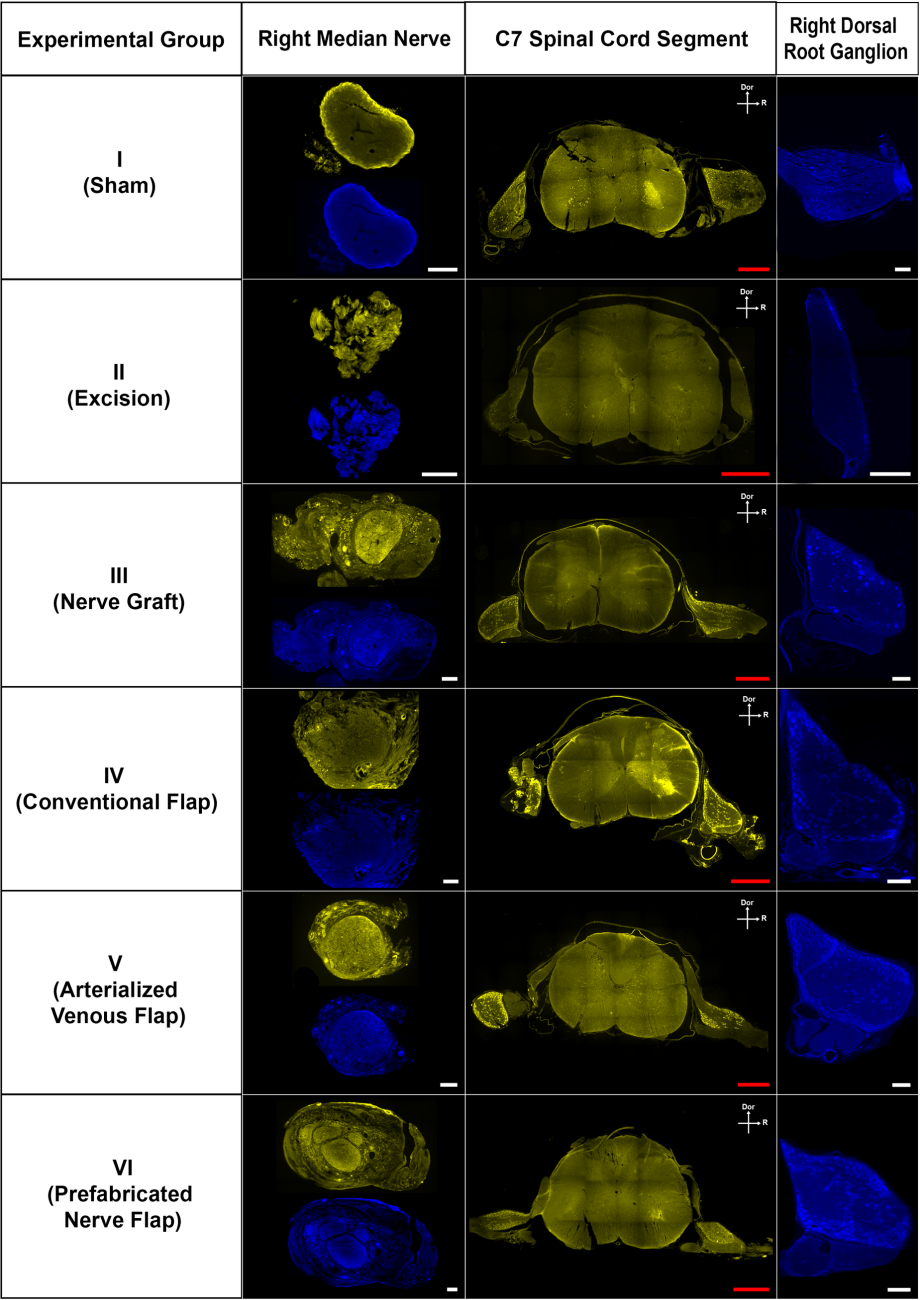
Fluorescence microscopy photographs of cross sections of the right median nerve proximally to the lesion, of the C7 spinal cord segment, and of the C7 the right dorsal root ganglion in the different experimental groups. Dor, dorsal; R, right Red calibration bar = 1 mm White calibration bar = 100 μm.

**Fig 20:**
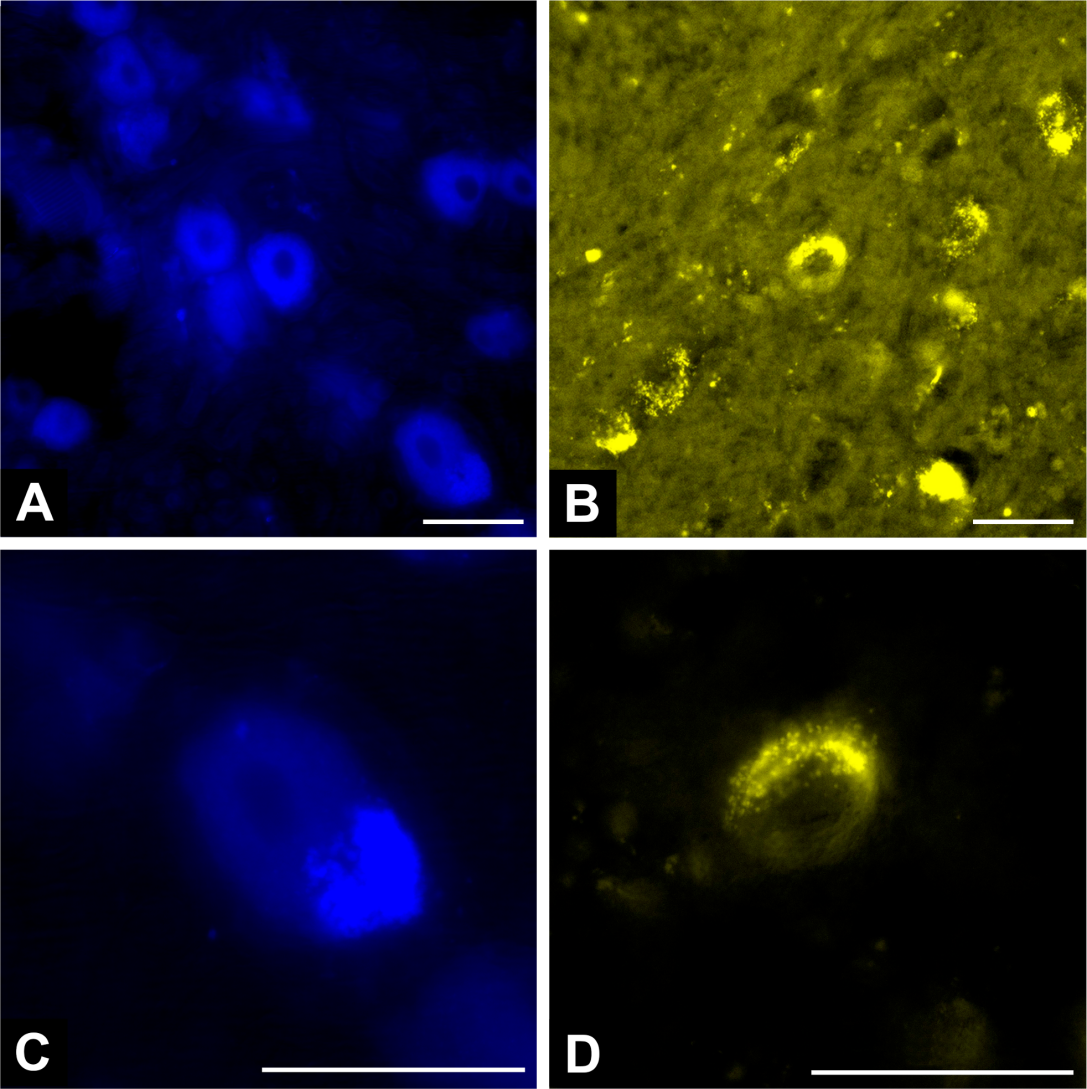
Typical high amplification fluorescence microscopy photographs of cross sections of the C7 the right dorsal root ganglion (**A** and **C**) showing ganglion cells stained with the True Blue® tracer and of motoneurons in the ventral horn of the spinal cord stained with the lucifer yellow (LY) ® tracer (**B** and **D**). Intracytoplasmic inclusions of these two markers are clearly visible in a rat of the Sham group. Calibration bar = 100 μm.

**Fig 21:**
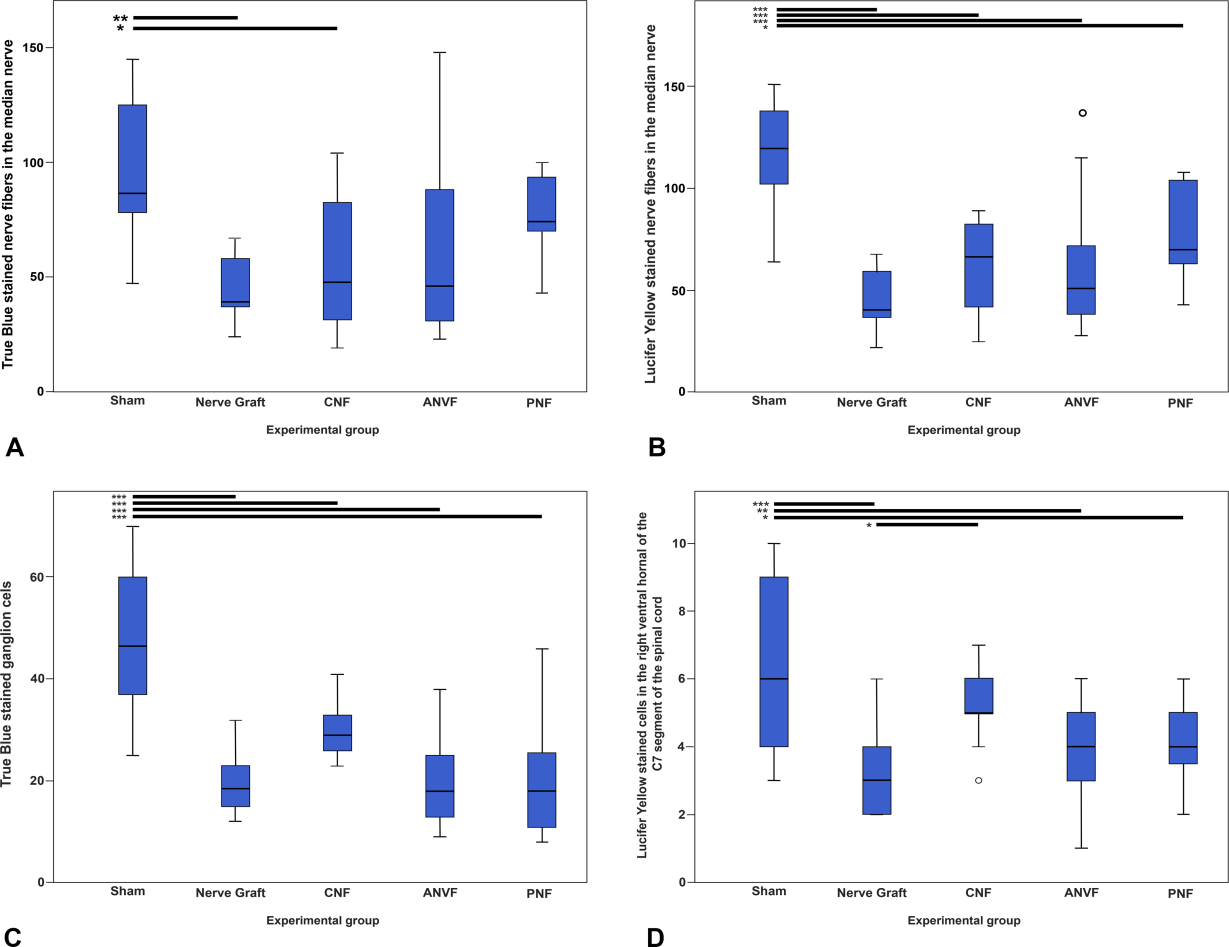
Semi quantitative evaluation of retrograde marking of the right median nerve proximally to the lesion site, of the right C7 dorsal ganglion and of the right ventral horn of the spinal cord at the C7 level in the different experimental groups. (A) Average number of True Blue diaceturate stained fibers in the right median nerve proximally to the repair site. (B) Average number of Lucifer Yellow CH dilithium stained fibers in the right median nerve proximally to the repair site. (C) Average number of True Blue diaceturate stained ganglion cells in the right C/ dorsal ganglion. (D) Average number of Lucifer Yellow CH dilithium stained cells in the ventral horn of the C7 spinal cord segment. Vertical lines represent 95% confidence intervals. Horizontal lines in the upper portion of the figure indicate statistically significant differences between experimental groups (p<0.05). *, p<0.05; **, p<0,01; ***, p<0.001.

Lucifer Yellow and True Blue were seen reaching the proximal MN, the C7 dorsal root ganglion and the ventral horn of the C7 spinal cord segment in all rats in the Sham, NG, CNF, ANVF and PNF groups (**Figs 19** and **20**). In the Excision group, although there was some degree of auto fluorescence, no clear intracytoplasmic markers were observed. Semi quantitative evaluation revealed a higher number of stained cells in the Sham group in all the regions studied (**Fig 21A** to **21D; S4 Table**). The CNF group presented a higher expression of fluorescent markers at all these locations than the NG group. However, this difference was statistically significant only for the Lucifer Yellow staining of the cells in the ventral horn of the spinal cord (p = 0.045; **Fig 21D**). There were no significant differences between the NG group and the ANVF and the PNF groups regarding fluorescence staining.

### Multiple correlations were found between functional motor variables and neurophysiological and histomorphometric variables

Time to recovery of grasping in the operated limb was positively correlated with the neurological threshold, and with the motor threshold. Time to recovery of grasping was negatively correlated with the following parameters: CMAP amplitude; FCR weight; maximal isometric wrist flexion strength; AUC in the strength x time graph; number of MN nerve fibers; number of MN acetylcholinesterase positive fibers; number of MN peripherin positive fibers; number of Lucifer Yellow positive fibers in the MN; number of True Blue marked DRG cells; and number of Lucifer Yellow marked neurons in the ventral horn of the spinal cord (**S5 Table**).

Similarly, FCR weight was positively correlated with the following variables: maximal isometric wrist flexion strength; AUC in the strength x time graph; CMAP amplitude; MN nerve area cross sectional area; number of MN nerve fibers; number of MN acetylcholinesterase positive fibers; number of MN peripherin positive fibers; number of MN acetylcholinesterase negative and peripherin negative fibers; vascular density in the reconstructed nerve gap; number of Lucifer Yellow positive fibers in the MN; number of True Blue stained DRG cells; and number of Lucifer Yellow marked neurons in the ventral horn of the spinal cord (**S5 Table**).

In opposition, FCR weight was negatively correlated with the neurological threshold, and with the motor threshold (**S5 Table**).

Additionally, maximal isometric wrist flexion strength was positively correlated with the following variables: CMAP amplitude; MN cross sectional area; number of MN nerve fibers; number of MN acetylcholinesterase positive fibers; number of Lucifer Yellow stained fibers in the MN; number of True Blue marked DRG cells; and number of Lucifer Yellow positive neurons in the ventral horn of the spinal cord (**S5 Table**).

Maximal isometric wrist flexion strength was negatively correlated with the neurological threshold; and with the motor threshold (**S5 Table**).

AUC in the strength x time graph was positively correlated with CMAP amplitude; the number of MN acetylcholinesterase positive fibers; the number of Lucifer Yellow stained fibers in the MN, and with the number of True Blue marked DRG (**S5 Table**).

AUC in the strength evaluation graph was negatively correlated with the motor threshold.

Velocity in the inclined ladder on D90 was positively correlated with the following assessments: FCR weight; maximal isometric wrist flexion strength; CMAP amplitude; MN cross sectional area; number of MN nerve; number of MN acetylcholinesterase positive fibers; number of MN peripherin positive fibers; and number of True Blue positive fibers in the MN (**S5 Table**).

Rats that did not present radial deviation in the walking tracks fared better that rats did present radial deviation in the following parameters: FCR weight (37.59% ± 16.27% vs. 84.25% ± 18.72%; p<0.001), maximal isometric flexion force (87.75% ± 62.36% vs. 27.47% ± 13.35%; p<0.001), MN cross section area (98.79% ± 22.10% vs. 66.79% ± 29.42%; p<0.001), total number of MN nerve fibers (87.43% ± 33.14% vs. 23.40% ± 37.77%; p<0.001); and number of peripherin stained fibers (88.08% ± 33.03% vs. 53.57% ± 41.28%; p = 0.021).

### Multiple correlations were found between nociception assessment and functional motor, neurophysiological and histomorphometric variables

Nociception evaluation by the pin prick test on D90 was positively correlated with velocity in the inclined ladder on D90, with CMAP amplitude, with MN cross section area, with the number of MN nerve fibers, with the number of MN acetylcholinesterase positive fibers, with the number of MN peripherin positive fibers, with the number of True Blue stained fibers in the MN, with the number of Lucifer Yellow stained fibers in the MN, with the number of stained DRG cells, and even with the number of Lucifer Yellow positive neurons in the ventral horn of the spinal cord. Finally, nociception evaluation by the pin prick test on D90 was negatively correlated the neurological threshold (**S6 Table**).

## Discussion

The authors believe that of the main merits of the present work was to apply in a same model of peripheral nerve gap and local ischemia, various autologous reconstructive techniques, in order to obtain more homogenous results. In fact, it is commonly accepted that it is difficult to conciliate the highly diverse experimental evidence, due to the various animal species tested, the multiple anatomical regions used, the inclusion or not of local ischemia, the different parameters evaluated, and the heterogenous follow-up time.[31] Additionally, to the best of the authors’ knowledge, this is the largest series in the literature comparing different autologous techniques of MN gap reconstruction in the rat, in the presence of local ischemia.[93–95]

Overall, the results of this article seem to lend to support to the notion that vascularized nerve conduits in an ischemic environment lead to a more rapid and complete recovery. In fact, for all the parameters assessed, the groups using vascularized nerve conduits presented at least as good result of NG, and, in many circumstances, they ensured a better result that the latter option. For example, the Grasping Test revealed faster and more complete motor recovery in the CNF and in the ANVF groups than in the NG group. Likewise, the Pin prick test showed better nociceptive recovery in the CNF and ANVF groups than in the NG group. Walking Track Analysis revealed that the rate of radial deviation was lower in groups in which a vascularized nerve conduit was used. ENMG assessment revealed a lower motor stimulation threshold in the CNF and ANVF than in the NG group. Moreover, CNF and the ANVF presented better FCR muscle weight than the NG group. Additionally, histomorphometric evaluation of the distal aspect of the MN showed a tendency to worse results in the NG group. In fact, CNF presented a higher MN cross section area, higher total number of nerve fibers, as well as higher number of peripherin positive and acetylcholinesterase negative and peripherin negative nerve fibers. Finally, histological characterization of the nerve conduits revealed greater architectural disorganization in the nerve graft conduit. The CNF group presented a higher expression of fluorescent markers at all these locations than the NG group.

Koshima *et al*. in 1985 had demonstrated that in the sciatic nerve of rats, CNFs yielded better results than NGs in reconstructing nerve defects in scarred regions after previous burns.[96, 97] Gu *et al*. in 1985 presented a study on New Zealand rabbits showing that CNFs were superior to nerve grafts for reconstructing a 20-mm MN defect even in favorable local conditions.[98] These data were soon validated in experiments performed in the same species by different authors.[99] However, subsequently, other authors concluded that nerve flaps did not benefit in reconstructing nerve defects in the context of normal perfusion in a rabbit model, suggesting that nerve flap reconstruction may be more beneficial in conditions of local ischemia.[31]

Notwithstanding, there is experimental evidence that CNFs, having a blood supply of its own, guarantee a better survival of Schwann cells, and are more efficiently permeated by macrophages, which will remove myelin fragments from degenerated axons.[100] Overall, these processes maintain a better architecture of the nerve conduit, promoting nerve regeneration.[23, 99–101] Blood supply to nerve conduits seems to be particularly critical in conditions of local ischemia, namely in regions of intense fibrosis, or prior radiotherapy.[23, 99–101] These experimental findings have been corroborated clinically. In fact, it is well established that good perfusion of the nerve repair zone is mandatory to ensure a good functional outcome. [2, 3, 102, 103]

Interestingly, in this work, CNFs and ANVFs were, for most of the assessed variables, comparable.[98] Vargel *et al*. using a femoral nerve model of ischemia in the rat showed that ANVFs presented superior results to NGs in nerve gap reconstruction.[29, 30] In fact, Townsend *et al*. had already demonstrated a faster rate of axonal elongation in ANVFs executed in the hindlimb of 15 greyhound dogs compared to NGs.[20]

The PNF group did not present as good results as the CNF and the ANVF groups. This may be due to the fact that the sciatic nerve not only is larger than the MN, but it is also polyfascicular, composed of motor, sensory and mixed fascicles.[47, 48, 104] The MN of the rat is, at the arm level, monofascicular (**Fig 17**).[105] These morphometric differences may have led to a poor correspondence between motor and sensory axons, which, in turn, may have been responsible for inferior results in the PNF group comparatively to the other groups using vascularized nerve conduits.[106] These data contrast with those presented in the report of Karcher *et al*., in which an arteriovenous fistula was used to produce a PNF involving the femoral nerve of the rat. This PFN provided better results in the reconstruction of a fascicle of the sciatic nerve of the rat than those obtained with an homologous NG.[33]

The authors used the concept of an enriched housing environment to better simulate the clinical condition of a patient submitted to a peripheral nerve repair surgery and offered postoperative physiotherapy.[107–109] This concept, which was coined in the 1940s, generally refers to keeping experimental animals in groups inside large cages, which are equipped with miscellaneous objects, namely running wheels, logs, and toys.[107–109] Enriched housing provides experimental animals with continuous and voluntary physiotherapy, which, according to various authors, promotes peripheral nerve regeneration and functional recovery [110–113], while minimizing the prevalence of neuropathic pain and autotomy.[107, 108, 112] Auto-mutilation, in turn, frequently makes further data collection from the affected limb impossible. Hence, enriched housing not only reduces animal suffering, but also minimizes the number of experimental animals needed to maintain a previously set statistical power.[107]

Interestingly, a consequence of the regular exercise was that from the beginning of the experiment, rats weighed less than their sedentary counterparts. [114, 115] Nevertheless, animal weight gain throughout the experiment was significant and similar in all groups (**Table 2**).

No significant differences were observed in the thermographic pattern of the different experimental groups 100 days after surgery (**Fig 11**). However, several authors have reported higher skin temperature in the territory of recently severed nerves, putatively related to the loss of activity of the sympathetic fibers contained in these nerves, leading to cutaneous vasodilation and consequently local increase in blood supply and, ultimately higher temperature.[70, 116, 117] Nevertheless, similarly to our findings, this association has not been demonstrated in cases of longstanding lesions.[118] For example, Sacharuk *et al*. have demonstrated a normalization of skin temperature in the hindlimb 21 days after a crush injury to the sciatic nerve.[118] In contrast, Kambiz *et al*. have shown that in streptozotocin induced diabetic rats, IRT was a non-invasive method that allowed an earlier diagnosis of peripheral neuropathy compared to classical methods, namely pin prick and ENMG evaluations.[119] However, all these works have used the sciatic nerve of the rat.[69, 70, 118–120] As far as the authors could determine, this paper describes for the first time the use of IRT in the realm of peripheral nerve regeneration in the rat’s forelimb.

Notwithstanding, the authors must concede that despite having strictly adhered to the available recommendations for performing IRT, this technique may be affected by several biases.[68, 69, 118, 120–122] Hence, further research in this area is warranted.[122, 123] In particular, it would be interesting to use an IRT camera with a higher resolution and a shorter minimal focus distance, in order to assess the usefulness of IRT to non-invasively evaluate reinnervation in the MN territory of the rat.

Remarkably, in this work multiple correlations were found between functional tests and neurophysiological and histomorphometric variables. In particular, an interesting finding of this study was the identification of radial deviation in forepaw prints of the rats with poorer outcomes in the following variables: FCR, maximal isometric flexion force, MN cross section area, total number of MN nerve fibers, and number of peripherin stained fibers. Thus, radial deviation of forepaws imprints may be of interest as surrogate marker of MN lesion.

The underlying mechanism for radial deviation may be the denervation, atrophy and shortening of the muscles of the forepaw of the rat innervated by the MN, generating a situation similar to the median claw hand observed in humans.[124, 125] In particular, shortening of the flexor carpi radialis (**Fig 16**) may accentuate the radial deviation of the paw (the rats contact the ground with their hands in the pronated position). Furthermore, the atrophy of the thenar and of the first three lumbrical muscles may also contribute to promote radial deviation (i.e., medial deviation) as well as to decrease the angle between the second and fourth digital rays. Nonetheless, further studies are required to confirm or dismiss this hypothesis. In particular, it would have been interesting to study the histology of the muscles of rats presenting radial deviation and comparing these findings with those of rats without radial deviation.

### Study limitations section

The mortality rate registered during the experimental procedure was significant, being higher in the PNF group than in the remaining experimental groups (60% versus 21%; p<0.05). This was probably due to the greater technical difficulty associated with creating a pre-fabricated nerve flap, requiring a challenging surgical procedure and the maintenance of a high throughput arterial-venous fistula. This, in turn, was the probable cause of the groin hematomas observed at the necropsy exam in 10 of the rats in the PNF group. These complications do not differ significantly from those reported in Cavada’s original description of the technique. According to these authors, only 73% of rats survived uneventfully to have their pre-fabricated nerve flap transferred to the arm region.[32] In fact, it has been long recognized that the number of rats dying in complex surgical experiments is frequently significant.[35, 126, 127] Similarly to other authors, in order to minimize the effect of attrition, it was decided beforehand to use a relatively large number of rats in each experimental group.[35, 127] Nevertheless, the relatively high mortality observed in this experiment, occurring randomly in the different groups, most likely decreased statistical power, without increasing the risk of false positive results.[128]

The authors opted to grade the grasping test using an ordinal scale rather than using a continuous one, in a similar fashion to that recently described by Stöβel *et al*.[57] In fact, it has been recently argued that the attribution of continuous numeric values to this test may be affected by several biases, namely:

- The absolute values may be significantly dependent on the strength the researcher applies while pulling the rodent’s tail.[129]
- It is difficult to dissociate the strength produced by the digital flexor muscles (exclusively innervated by the MN in the rat and the object of this test[48]) from the strength produced by the wrist flexors, which include the flexor carpi ulnaris that is innervated by the ulnar nerve.[56] This probably explains why, in the present study, 90 days after surgery, blinded observers considered that 35.3% of the rats in the Excision Group, presented flexion of the fingers in the operated limb, although not against resistance, attributing a grade 1 score to these rats (**Figs 16** and **17**)
- To obtain a numeric value, the contralateral paw has to be paralyzed in the territory of the median nerve. Two attain this goal, two strategies have been devised. Bertelli *et al*. proposed to immobilize the contra-lateral paw by wrapping it round with adhesive tape.[39] However, this not only is stressful to the animal, but also causes the rat to be frequently more concentrated in freeing the wrapped healthy limb than in grasping the grid with the operated forepaw.[56] Other authors have proposed to transect the contra-lateral median nerve.[56] Nevertheless, this method prevents the use of the contralateral paw as a convenient healthy control.

Taking into account all these potential sources of biases, the authors opted to use an ordinal scale similar to the Medical Research Council Scale commonly used to grade muscle strength in humans, as described in the Methods Section.[57, 130]

Noteworthily, the trends in motor and sensory recovery between the different experimental groups were not homogenous for all the outcome variables assessed. However, the underlying mechanisms of peripheral nerve recovery are known to be complex and time-dependent, involving many issues affecting neuron survival, proximal axon regeneration, synaptogenesis, recovery of the denervated motor and sensory targets, as well as cerebral plasticity.[11, 103, 131, 132]

Apart from radial deviation, walking track analysis failed to provide consistent differences between groups. However, other authors have also found that single MN lesions in the rat frequently do not produce consistent changes in the walking pattern.[45] Moreover, other authors have argued that pawprint analysis is more useful for crushing nerve lesions than for segmental nerve defect reconstruction.[11] Furthermore, it has also been shown that walking track analysis does not always correlate with muscle recovery.[133]

Regarding the choice of animal model, the rat sciatic nerve is arguably the most used nerve in peripheral nerve research.[54, 134] Notwithstanding, in this work the authors decided to use the rat MN, as the latter may present various advantages relatively to the former.[45, 46] In fact, MN lesions seem to be associated with lesser incidence of joint contractures and auto-mutilation of the affected limb.[45] Overall, rat welfare is more preserved with MN lesions than with sciatic nerve lesions.[54, 135] In addition, as the MN is shorter than the sciatic nerve, nerve recovery is observed sooner.[45, 134, 136–138] On top of this, most peripheral nerve lesions in the human species occur in the upper limb, further validating the use of this nerve in the rat.[3, 36, 139] Also, fine movement coordination in hand and finger movements is remarkably similar in rats and humans.[54, 140] Finally, recently, multiple standardized strategies have been introduced to assess motor and sensory recovery in the rat MN model, permitting an easier comparison of results.[40, 46, 47, 54, 65, 133, 141, 142]

Regarding the induction of a local ischemic environment, other authors have used a silicone barrier around the nerve repair zone in order to simulate a local ischemic medium.[30, 44] However, it may be argued that this model is not perfect, as silicone rods have been successfully used to reconstruct nerve defects in substitution of autologous conduits.[143, 144]

Also, the authors must concede that a major caveat of the present work is that rat peripheral nerves have much smaller cross-sectional areas than the homologous human structures. Theoretically, this should facilitate nerve revascularization, and promote better overall results in rats comparatively to humans.[30] In this sense, it would be useful to try to replicate the study herein described in larger animals. Furthermore, it is well established that reinnervation and functional recovery is more likely when nerve targets are closer to the repair zone.[131] The reasons for these are multiple: atrophy and fatty replacement of chronically denervated muscle, chronically denervated Schwann cells being less able to support regenerating axons, distal nerve histological disorganization increasing the likelihood of regenerating axons going into inappropriate endoneurial tubes and target organs (e.g. motor axons growing into endoneurial tubes connected to skin sensory organs or sensory axons growing into endoneurial tubes destined to motor plates).[131, 145] Moreover, under optimal conditions, in mammals, axonal elongation occurs at a rate of approximately 1–3 mm/day, limited by slow anterograde axonal transport.[3, 103, 131] This means that rats and humans have similar nerve regeneration speeds. Yet, rat’s nerves end organs are much closer to the place of nerve repair than in Man. Hence, the results of nerve repair are much faster in rats, which is convenient from an experimental point of view.[8, 96, 146, 147] Nevertheless, nerve recovery will probably be more complete than that observed in humans in similar circumstances.[3, 103, 148] To curb these biases, in the present study, the authors have restricted follow-up to 100 days. In fact, such follow-up time is used by most other researchers, in order to mitigate the effect of the exceptional neuroregenerative potential of rats.[93, 95] However, for all these reasons, the extrapolation of our results to the clinical setting should be made with caution. It would therefore be useful to try to replicate our findings in larger animals.

Another technical aspect that should be born in mind when comparing the results presented in this paper with those of other authors is that in the present work the excised MN segment was not inverted, as it is customary.[45, 49] The inversion of the nerve segment minimizes distal dispersion of the growing axons, maximizing the odds of these axons reaching their target organs, and ultimately leading to better functional recovery.[45, 49] In spite of this, the need of obtaining vascularized nerve conduits in this work precluded MN inversion. Still, this variable was the same in all experimental groups, maintaining internal consistency of results.

Additionally, rat sexual dimorphism in nerve regeneration should be taken into account.[54] Female rats present better nerve regeneration, presumably because of the beneficial effects of sex hormones.[54, 149–151] This fact leads many researchers to use female rats in peripheral nerve regeneration to maximize differences between experimental groups.[54] However, in humans, most injuries occur in males.[36, 152]

As other authors, in the present work we have used a morphological assessment of the blood supply to the reconstructed nerve segment, which was the *vasa nervorum* density.[153] Nevertheless, it would be interesting to precisely evaluate the perfusion in each of the nerve conduits used using other methods in future studies.[154] Quantitative microelectrode hydrogen clearance polarography, laser doppler flowmetry, autoradiography employing radionuclides, and microsphere embolization would be viable alternatives.[153]

The arterio-venous fistula used to produce PNFs could have been applied in the contralateral forelimb, to produce a PNF involving a MN segment homologous to the nerve defect. This could potentially have facilitated comparison of the different vascular patterns used in nerve flaps. Though, this option was not favored in the present study, as it would be technically vexing, and it would cause a major motor limitation in both forepaws, potentially compromising rats’ abilities to conduct their daily activities, such as feeding or grooming.

In this study, only the most commonly described unconventional perfusion nerve flap was used, the ANVF. Nevertheless, venous flow-through NFs have been shown to yield similar results to those obtained with ANVFs in a rat femoral nerve model.[29, 30] Further studies are warranted to confirm or dismiss this findings in the model used in the present study. Finally, it would have been interesting to include in this work a group using a rat MN allograft, as these conduits have been gaining increasing popularity in clinical practice.[10, 11, 24, 144] Noteworthily, Giusti *et al*. have recently demonstrated in a rat sciatic nerve model that blocking allograft vascularization from surrounding tissues was detrimental for motor recovery.[24] Thus, further studies are warranted in this field.

## Conclusion

CNFs and ANVFs produced a faster and more complete recovery than NGs in the reconstruction of a 10-mm-long median nerve gap in an ischemic environment in the Wistar rat. Although results obtained with CNFs were in most cases better than those of ANVFs, these differences were not statistically significant for most of the outcome variables.

## Supporting information

S1 Table Walking track analysis results throughout the experiment. **NG**, nerve graft; **CNF**, conventional nerve flap; **ANVF**, arterialized neurovenous flap; **PNF**, prefabricated nerve flap.
**D**, day after the beginning of the experiment.
Numeric variables are expressed as average ± standard deviation.
(DOCX)

S2 Table Infra-red thermography evaluation of the region of the forepays innervated by the median nerve 90 days postoperatively. **NG**, nerve graft; **CNF**, conventional nerve flap; **ANVF**, arterialized neurovenous flap; **PNF**, prefabricated nerve flap.
**D**, day after the beginning of the experiment.
Numeric variables are expressed as average ± standard deviation.
(DOCX)

S3 Table Histomorphometric evaluation of the right median nerve distally to the repair zone and of the vascular density in the middle portion of the reconstructed nerve defect in the different experimental groups. **NG**, nerve graft; **CNF**, conventional nerve flap; **ANVF**, arterialized neurovenous flap; **PNF**, prefabricated nerve flap.
**N/A**, non-applicable.
All parameters are expressed as percentages of the average contralateral values.
Numeric variables are expressed as average ± standard deviation.
(DOCX)

S4 Table Evaluation of retrograde marking of the right median nerve proximally to the lesion site, of the right C7 dorsal ganglion and of the right ventral horn of the spinal cord at the C7 level. **NG**, nerve graft; **CNF**, conventional nerve flap; **ANVF**, arterialized neurovenous flap; **PNF**, prefabricated nerve flap.
**N/A**, non-applicable.
All parameters are expressed as percentages of the average contralateral values.
Numeric variables are expressed as average ± standard deviation.
(DOCX)

S5 Table Summary of the correlations found between functional motor variables and neurophysiological and histomorphometric variables. CMAP, compound muscle action potentials; FCR, flexor carpi radialis; AUC, area under the curve; MN, median nerve; DRG, dorsal root ganglion.
(DOCX)

S6 Table Summary of the correlations found between nociception assessment 90 days after surgery and functional motor, neurophysiological and histomorphometric variables. D90, ninety days after surgery; CMAP, compound muscle action potentials; MN, median nerve; DRG, dorsal root ganglion.
(DOCX)
