# Supplementary material for: Reconstruction of a 10-mm-long median nerve gap in an ischemic environment using autologous conduits with different patterns of blood supply: A comparative study in the rat
Source: PLoS One. 2018 Apr 16;13(4):e0195692. doi: 10.1371/journal.pone.0195692 (PMC5902043; doi:10.1371/journal.pone.0195692)
Supplement: S1 Table — NG, nerve graft; CNF, conventional nerve flap; ANVF, arterialized neurovenous flap; PNF, prefabricated nerve flap. D, day after the beginning of the experiment. Numeric variables are expressed as average ± standard deviation. (DOCX) [file pone.0195692.s001.docx]

| **Parameter** | | **Sham group** | **Excision group** | **NG**  **group** | **CNF**  **group** | **ANVF group** | **PNF**  **group** | **Relevant**  **findings** |
| --- | --- | --- | --- | --- | --- | --- | --- | --- |
| **Stance factor**  **(%)** | **D30** | 97.02 ± 20.42 | 70.57 ± 18.01 | 70.91 ± 18.64 | 103.34 ± 22.69 | 88.72 ± 19.44 | 72.39 ± 40.35 | On D30 the CNF group presented a better normalized stance factor than the excision, NG and PNF groups (p<0.001) |
|  | **D45** | 100.65 ± 14.18 | 120.18 ± 40.80 | 77.85 ± 28.34 | 114.91 ± 21.08 | 100.93 ± 15.61 | 106.07 ± 10.93 |  |
|  | **D60** | 95.37 ± 14.98 | 87.47 ± 20.83 | 88.79 ± 16.99 | 88.79 ± 16.99 | 81.82 ± 22.12 | 86.66 ± 14.66 |  |
|  | **D75** | 98.75 ± 16.03 | 92.59 ± 24.66 | 77.08 ± 35.73 | 77.08 ± 35.73 | 87.24 ± 25.43 | 86.70 ± 17.38 |  |
|  | **D90** | 104.56 ± 11.47 | 85.63 ± 27.23 | 102.87 ± 19.10 | 85.77 ± 16.12 | 94.79 ± 15.80 | 112.55 ± 38.97 |  |
| **Print length**  **(%)** | **D30** | 98.24 ± 2.95 | 94.41 ± 3.31 | 88.18 ± 3.84 | 97.45 ± 3.45 | 93.38 ± 5.19 | 94.35 ± 5.19 | On D30 print length was higher in the CNF, ANVF and PNF groups than in the NG group on D30 (p<0.01) |
|  | **D45** | 99.19 ± 5.21 | 97.15 ± 5.36 | 91.11 ± 2.19 | 96.59 ± 3.02 | 95.52 ± 13.62 | 99.56 ± 4.00 |  |
|  | **D60** | 100.16 ± 4.44 | 98.45 ± 2.45 | 96.40 ± 2.85 | 97.46 ±4.39 | 94.79 ± 12.94 | 92.72 ± 4.55 |  |
|  | **D75** | 99.98 ± 5.19 | 97.21 ± 5.10 | 95.64 ± 3.21 | 95.29 ± 2.86 | 88.66 ± 16.80 | 98.93 ± 4.21 |  |
|  | **D90** | 100.00 ± 4.00 | 97.18 ± 4.49 | 93.63 ± 2.80 | 94.51 ± 1.92 | 88.70 ± 10.85 | 97.88 ± 2.13 |  |
| **Finger spread**  **(%)** | **D30** | 96.16 ± 10.83 | 85.58 ± 8.05 | 66.72 ± 8.75 | 94.71 ± 4.20 | 99.98 ± 14.70 | 89.15 ± 3.24 | On D30  the CNF, ANVF, and PNF groups presented better results than the NG group  (p<0.05) |
|  | **D45** | 94.21 ± 4.06 | 90.20 ± 9.69 | 79.64 ± 7.01 | 87.96 ± 7.68 | 95.87 ± 21.38 | 98.35 ± 10.43 |  |
|  | **D60** | 95.61 ± 5.28 | 89.70 ± 9.74 | 78.94 ± 11.59 | 89.99 ± 10.54 | 87.75 ± 19.14 | 91.37 ± 3.60 |  |
|  | **D75** | 111.47 ± 38.51 | 106.03 ± 45.96 | 86.89 ± 5.78 | 87.87 ± 7.67 | 86.31 ± 26.29 | 95.13 ± 5.03 |  |
|  | **D90** | 102.07 ± 10.52 | 93.57 ± 10.37 | 84.57 ± 12.18 | 88.11 ± 6.71 | 80.42 ± 23.16 | 99.63 ± 6.74 |  |
| **Intermediate finger spread (%)** | **D30** | 94.58 ± 12.14 | 89.42 ± 14.37 | 76.13 ± 18.89 | 89.09 ± 10.04 | 93.15 ± 13.70 | 81.21 ± 9.56 | On D30  the CNF, ANVF, and PNF groups presented better results than the NG group  (p<0.05) |
|  | **D45** | 89.93 ± 8.65 | 88.23 ± 14.16 | 77.92 ± 15.33 | 87.89 ± 14.93 | 91.15 ± 24.47 | 96.22 ± 8.38 |  |
|  | **D60** | 95.61 ± 5.28 | 89.70 ± 9.74 | 78.94 ± 11.59 | 89.99 ± 10.54 | 78.22 ± 24.92 | 88.14 ± 10.83 |  |
|  | **D75** | 102.32 ± 10.46 | 80.44 ± 26.49 | 85.18 ± 7.51 | 87.74 ± 15.62 | 78.67 ± 31.31 | 94.30 ± 12.43 |  |
|  | **D90** | 102.07 ± 10.52 | 93.57 ± 10.37 | 84.57 ± 12.18 | 88.11 ± 6.71 | 93.63 ± 6.34 | 91.53 ± 6.37 |  |

| **Parameter** | | **Sham group** | **Excision group** | **NG**  **group** | **CNF**  **group** | **ANVF group** | **PNF**  **group** | **Relevant**  **findings** |
| --- | --- | --- | --- | --- | --- | --- | --- | --- |
| **Stride length**  **(%)** | **D30** | 102.11 ± 6.80 | 102.86 ± 9.79 | 102.93 ± 8.39 | 109.47 ± 10.52 | 95.86 ± 4.09 | 106.26 ± 29.08 | No significant differences between experimental groups |
|  | **D45** | 105.44 ± 12.32 | 103.59 ± 10.12 | 109.39 ± 14.56 | 116.24 ± 13.17 | 111.60 ± 32.18 | 101.15 ± 5.96 |  |
|  | **D60** | 100.66 ± 10.50 | 101.05 ± 9.82 | 105.18 ± 7.52 | 100.69 ± 3.95 | 105.63 ± 6.43 | 114.53 ± 26.66 |  |
|  | **D75** | 100.14 ± 7.62 | 95.74 ± 12.45 | 100.67 ± 15.26 | 102.40 ± 12.81 | 99.84 ± 8.70 | 111.28 ± 11.85 |  |
|  | **D90** | 100.40 ± 10.31 | 97.58 ± 12.05 | 95.74 ± 9.62 | 107.22 ± 10.75 | 100.37 ± 10.35 | 114.25 ± 17.68 |  |
| **Base of support**  **(%)** | **D30** | 82.16 ± 38.70 | 77.42 ± 39.42 | 91.22 ± 23.90 | 149.03 ± 10.49 | 99.97 ± 56.78 | 87.93 ± 18.65 | No significant differences between experimental groups |
|  | **D45** | 74.38 ± 26.77 | 131.15 ± 58.52 | 82.24 ± 13.51 | 123.14 ± 47.25 | 108.52 ± 25.71 | 105.10 ± 47.60 |  |
|  | **D60** | 108.78 ± 42.96 | 108.13 ± 20.38 | 107.00 ± 22.48 | 109.57 ± 18.87 | 75.00 ± 33.74 | 112.26 ± 64.79 |  |
|  | **D75** | 87.68 ± 32.12 | 70.16 ± 26.24 | 138.07 ± 75.83 | 84. 88 ± 20.40 | 173.19 ± 87.47 | 135.91 ± 84.45 |  |
|  | **D90** | 100.63 ± 24.07 | 106.52 ± 27.14 | 111.80 ± 67.69 | 135.57 ± 53.80 | 95.20 ± 28.02 | 127.17 ± 61.37 |  |
| **Presence of radial deviation (%)** | **D30** | 0 | 94.1 | 100 | 40 | 13.3 | 0 | For each time point, the rate of radial deviation was lower in groups in which a vascularized nerve conduit was used compared to the NG group (p<0.001) |
|  | **D45** | 0 | 100 | 100 | 20 | 13.3 | 0 |  |
|  | **D60** | 0 | 100 | 100 | 0 | 20 | 0 |  |
|  | **D75** | 0 | 100 | 70 | 0 | 20 | 0 |  |
|  | **D90** | 0 | 100 | 30 | 0 | 20 | 0 |  |

**Supplemental Table 1.** Walking track analysis results throughout the experiment.

**NG**, nerve graft; **CNF**, conventional nerve flap; **ANVF**, arterialized neurovenous flap; **PNF**, prefabricated nerve flap

**D**, day after the beginning of the experiment

Numeric variables are expressed as average ± standard deviation.
