# Supplementary material for: Reconstruction of a 10-mm-long median nerve gap in an ischemic environment using autologous conduits with different patterns of blood supply: A comparative study in the rat
Source: PLoS One. 2018 Apr 16;13(4):e0195692. doi: 10.1371/journal.pone.0195692 (PMC5902043; doi:10.1371/journal.pone.0195692)
Supplement: S2 Table — NG, nerve graft; CNF, conventional nerve flap; ANVF, arterialized neurovenous flap; PNF, prefabricated nerve flap. D, day after the beginning of the experiment. Numeric variables are expressed as average ± standard deviation. (DOCX) [file pone.0195692.s002.docx]

| **Parameter** | **Sham group** | **Excision group** | **NG group** | **CNF group** | **ANVF group** | **PNF group** | **Relevant**  **findings** |
| --- | --- | --- | --- | --- | --- | --- | --- |
| **Forepaw average temperature**  **(%)** | 99.4 ± 5.9 | 101.7 ± 7.1 | 104.9 ± 13.7 | 101.8 ± 8.0 | 103.6 ± 7.2 | 109.0 ± 10.8 | No significant differences between experimental groups |
| **Forepaw minimal temperature**  **(%)** | 100.45 ± 5.49 | 102.25 ± 7.04 | 105.47 ± 14.34 | 102.45 ± 8.39 | 104.36 ± 7.83 | 108.61 ± 11.58 | No significant differences between experimental groups |
| **Forepaw maximal temperature**  **(%)** | 98.86 ± 6.43 | 99.97 ± 6.41 | 101.44 ± 6.94 | 100.42 ± 6.74 | 100.63 ± 3.17 | 107.03 ± 5.04 | No significant differences between experimental groups |

**Supplemental Table 2.** Infra-red thermography evaluation of the region of the forepays innervated by the median nerve 90 days postoperatively.

**NG**, nerve graft; **CNF**, conventional nerve flap; **ANVF**, arterialized neurovenous flap; **PNF**, prefabricated nerve flap

**D**, day after the beginning of the experiment

Numeric variables are expressed as average ± standard deviation.
