# Supplementary material for: Reconstruction of a 10-mm-long median nerve gap in an ischemic environment using autologous conduits with different patterns of blood supply: A comparative study in the rat
Source: PLoS One. 2018 Apr 16;13(4):e0195692. doi: 10.1371/journal.pone.0195692 (PMC5902043; doi:10.1371/journal.pone.0195692)
Supplement: S3 Table — NG, nerve graft; CNF, conventional nerve flap; ANVF, arterialized neurovenous flap; PNF, prefabricated nerve flap. N/A, non-applicable. All parameters are expressed as percentages of the average contralateral values. Numeric variables are expressed as average ± standard deviation. (DOCX) [file pone.0195692.s003.docx]

| **Parameter** | **Sham group** | **Excision group** | **NG**  **group** | **CNF**  **group** | **ANVF group** | **PNF group** | **Relevant**  **findings** |
| --- | --- | --- | --- | --- | --- | --- | --- |
| **Cross section area**  **(%)** | 106.59 ± 27.45 | 54.67 ± 26.55 | 72.42 ± 30.28 | 93.38 ± 10.87 | 82.44 ± 15.77 | 109.42 ± 19.86 | Higher in the PNF group than in the NG group (p<0.05) |
| **Total number of fibers**  **(%)** | 101.76 ± 5.30 | N/A | 56.88 ± 17.85 | 97.91 ± 50.72 | 67.71 ± 18.58 | 70.33 ± 9.31 | Higher in the CNF group than in the NG group (p<0.01) |
| **Acetylycholinesterase positive nerve fibers**  **(%)** | 106.94 ± 17.65 | N/A | 40.90 ± 5.54 | 49.67 ± 8.61 | 49.67 ± 8.61 | 47.07 ± 10.82 | No significant differences between experimental groups |
| **Peripherin positive nerve fibers**  **(%)** | 101.64 ± 5.53 | N/A | 56.90 ± 17.83 | 101.45 ± 49.23 | 101.45 ± 49.23 | 73.03 ± 11.49 | Numbers of peripherin positive and acethyl-cholinesterase negative and peripherin negative fibers were higher in the CNF and in the Sham groups than in the NG group (p<0.001) |
| **Acethylcholinesterase negative and peripherin negative nerve fibers**  **(%)** | 95.58 ± 10.76 | N/A | 61.02 ± 21.59 | 126.28 ± 78.51 | 73.20 ± 22.36 | 75.03 ± 11.61 |  |
| **Vascular density in the middle portion of the reconstructed nerve segment**  **(%)** | 335.98 ± 155.89 | N/A | 126.70 ± 50.92 | 249.67 ± 62.73 | 310.51 ± 188.86 | 385.04 ± 225.80 | Higher in the PNF group than in the NG group (p<0.05) |

**Supplemental Table 3.** Histomorphometric evaluation of the right median nerve distally to the repair zone and of the vascular density in the middle portion of the reconstructed nerve defect in the different experimental groups

**NG**, nerve graft; **CNF**, conventional nerve flap; **ANVF**, arterialized neurovenous flap; **PNF**, prefabricated nerve flap

**N/A**, non-applicable

All parameters are expressed as percentages of the average contralateral values.

Numeric variables are expressed as average ± standard deviation.
