# Supplementary material for: Reconstruction of a 10-mm-long median nerve gap in an ischemic environment using autologous conduits with different patterns of blood supply: A comparative study in the rat
Source: PLoS One. 2018 Apr 16;13(4):e0195692. doi: 10.1371/journal.pone.0195692 (PMC5902043; doi:10.1371/journal.pone.0195692)
Supplement: S4 Table — NG, nerve graft; CNF, conventional nerve flap; ANVF, arterialized neurovenous flap; PNF, prefabricated nerve flap. N/A, non-applicable. All parameters are expressed as percentages of the average contralateral values. Numeric variables are expressed as average ± standard deviation. (DOCX) [file pone.0195692.s004.docx]

| **Parameter** | **Sham**  **group** | **NG**  **group** | **CNF**  **group** | **ANVF**  **group** | **PNF**  **group** | **Relevant**  **findings** |
| --- | --- | --- | --- | --- | --- | --- |
| **True Blue stained axons in the median nerve** | 96.10 ± 30.70 | 44.00 ± 14.29 | 55.58 ± 30.06 | 61.93 ± 40.55 | 77.25 ± 18.50 | Higher average value in the Sham group than in the NG and CNF groups (p<0.05) |
| **Diamidino Yellow stained axons in the median nerve** | 115.70 ± 28.49 | 45.10 ± 14.40 | 63.58 ± 21.88 | 63.07 ± 33.22 | 78.12 ± 24.19 | No significant differences in the experimental groups |
| **Stained ganglion cells** | 48.20 ± 13.95 | 19.60 ± 6.36 | 29.92 ± 5.19 | 20.13 ± 8.49 | 20.38 ± 12.47 | No significant differences in the experimental groups |
| **Stained ventral horn cells** | 6.50 ± 2.59 | 3.20 ± 1.23 | 5.25 ± 1.14 | 3.80 ± 1.47 | 4.12 ± 1.25 | The CNF group presented a higher average value than the NG group (p=0.045) |

**Supplemental Table 4.** Evaluation of retrograde marking of the right median nerve proximally to the lesion site, of the right C7 dorsal ganglion and of the right ventral horn of the spinal cord at the C7 level.

**NG**, nerve graft; **CNF**, conventional nerve flap; **ANVF**, arterialized neurovenous flap; **PNF**, prefabricated nerve flap

**N/A**, non-applicable

All parameters are expressed as percentages of the average contralateral values.

Numeric variables are expressed as average ± standard deviation.
