# Supplementary material for: Reconstruction of a 10-mm-long median nerve gap in an ischemic environment using autologous conduits with different patterns of blood supply: A comparative study in the rat
Source: PLoS One. 2018 Apr 16;13(4):e0195692. doi: 10.1371/journal.pone.0195692 (PMC5902043; doi:10.1371/journal.pone.0195692)
Supplement: S5 Table — CMAP, compound muscle action potentials; FCR, flexor carpi radialis; AUC, area under the curve; MN, median nerve; DRG, dorsal root ganglion. (DOCX) [file pone.0195692.s005.docx]

| **Motor variable** | **Neurophysiological and and histomorphometric variable** | **Pearson’s correlation coefficient** | **P value** |
| --- | --- | --- | --- |
| Time to recovery of grasping | Neurological threshold | 0.484 | <0.001 |
|  | Motor threshold | 0.735 | <0.001 |
|  | CMAP amplitude | -0.611 | <0.001 |
|  | FCR weight | -0.769 | <0.001 |
|  | Maximal isometric wrist flexion strength | -0.571 | <0.001 |
|  | AUC in the strength x time graph | -0.324 | 0.003 |
|  | Number of MN nerve fibers | -0.464 | <0.001 |
|  | Number of MN acetylcholinesterase positive fibers | -0.718 | <0.001 |
|  | Number of MN peripherin positive fibers | -0.393 | 0.004 |
|  | Number of Lucifer Yellow positive fibers in the MN | -0.409 | 0.002 |
|  | Number of True Blue marked DRG cells | -0.511 | <0.001 |
|  | Number of Lucifer Yellow marked neurons in the ventral horn of the spinal cord | -0.393 | 0.004 |
| FCR weight | Maximal isometric wrist flexion strength | 0.548 | <0.001 |
|  | AUC in the strength x time graph | 0.288 | 0.009 |
|  | CMAP amplitude | 0.724 | <0.001 |
|  | MN nerve area cross sectional area | 0.486 | <0.001 |
|  | Number of MN nerve fibers | 0.644 | <0.001 |
|  | Number of MN acetylcholinesterase positive fibers | 0.624 | <0.001 |
|  | Number of MN peripherin positive fibers | 0.454 | 0.001 |
|  | Number of MN acetylcholinesterase negative and peripherin negative fibers | 0.294 | 0.038 |
|  | Vascular density in the reconstructed nerve gap | 0.337 | 0.019 |
|  | Number of Lucifer Yellow positive fibers in the MN | 0.356 | 0.008 |
|  | Number of True Blue stained DRG cells | 0.418 | 0.002 |
|  | Number of Lucifer Yellow marked neurons in the ventral horn of the spinal cord | 0.352 | 0.008 |
|  | Neurological threshold | -0.617 | <0.001 |
|  | Motor threshold | -0.803 | <0.001 |
| Maximal isometric wrist flexion strength | CMAP amplitude | 0.434 | <0.001 |
|  | MN cross sectional area | 0.292 | 0.024 |
|  | Number of MN nerve fibers | 0.378 | 0.003 |
|  | Number of MN acetylcholinesterase positive fibers | 0.639 | <0.001 |
|  | Number of Lucifer Yellow stained fibers in the MN | 0.357 | 0.007 |
|  | Number of True Blue marked DRG cells | 0.396 | 0.003 |
|  | Number of Lucifer Yellow positive neurons in the ventral horn of the spinal cord | 0.521 | <0.001 |
|  | Neurological threshold | -0.324 | 0.003 |
|  | Motor threshold | -0.511 | <0.001 |

| **Motor variable** | **Neurophysiological and and histomorphometric variable** | **Pearson’s correlation coefficient** | **P value** |
| --- | --- | --- | --- |
| AUC in the strength x time graph | CMAP amplitude | 0.428 | <0.001 |
|  | Number of MN acetylcholinesterase positive fibers | 0.458 | 0.001 |
|  | Number of Lucifer Yellow stained fibers in the MN | 0.305 | 0.023 |
|  | Number of True Blue marked DRG cells | 0.312 | 0.021 |
|  | Motor threshold | -0.321 | 0.003 |
| Velocity in the inclined ladder on D90 | FCR weight | 0.547 | <0.001 |
|  | Maximal isometric wrist flexion strength | 0.248 | <0.024 |
|  | CMAP amplitude | 0.428 | <0.001 |
|  | MN cross sectional area | 0.302 | 0.012 |
|  | Number of MN nerve fibers | 0.474 | <0.001 |
|  | Number of MN acetylcholinesterase positive fibers | 0.434 | 0.002 |
|  | Number of MN peripherin positive fibers | 0.334 | 0.012 |
|  | Number of True Blue positive fibers in the MN | 0.305 | 0.012 |

**Supplemental Table 5.** Summary of the correlations found between functional motor variables and neurophysiological and histomorphometric variables.

CMAP, compound muscle action potentials; FCR, flexor carpi radialis; AUC, area under the curve; MN, median nerve; DRG, dorsal root ganglion.
