# Supplementary material for: Reconstruction of a 10-mm-long median nerve gap in an ischemic environment using autologous conduits with different patterns of blood supply: A comparative study in the rat
Source: PLoS One. 2018 Apr 16;13(4):e0195692. doi: 10.1371/journal.pone.0195692 (PMC5902043; doi:10.1371/journal.pone.0195692)
Supplement: S6 Table — D90, ninety days after surgery; CMAP, compound muscle action potentials; MN, median nerve; DRG, dorsal root ganglion. (DOCX) [file pone.0195692.s006.docx]

| **Nociceptive evaluation on D90** | **Neurophysiological and and histomorphometric variable** | **Pearson’s correlation coefficient** | **P value** |
| --- | --- | --- | --- |
| Pin prick test | Velocity in the inclined ladder on D90 | 0.550 | <0.001 |
|  | CMAP amplitude | 0.589 | <0.001 |
|  | MN cross section area | 0.422 | 0.001 |
|  | Number of MN nerve fibers | 0.614 | <0.001 |
|  | Number of MN acetylcholinesterase positive fibers | 0.316 | 0.011 |
|  | Number of MN peripherin positive fibers | 0.348 | 0.012 |
|  | Number of True Blue stained fibers in the MN | 0.295 | 0.029 |
|  | Number of Lucifer Yellow stained fibers in the MN | 0.293 | 0.030 |
|  | Number of stained DRG cells | 0.274 | 0.043 |
|  | Number of Lucifer Yellow positive neurons in the ventral horn of the spinal cord | 0.339 | 0.011 |
|  | Neurological threshold | -0.561 | 0.003 |

**Supplemental Table 6.** Summary of the correlations found between nociception assessment 90 days after surgery and functional motor, neurophysiological and histomorphometric variables.

D90, ninety days after surgery; CMAP, compound muscle action potentials; MN, median nerve; DRG, dorsal root ganglion.
